# c-Abl-TWIST1 Epigenetically Dysregulate Inflammatory Responses during Mycobacterial Infection by Co-Regulating Bone Morphogenesis Protein and miR27a

**DOI:** 10.3389/fimmu.2018.00085

**Published:** 2018-02-01

**Authors:** Kasturi Mahadik, Praveen Prakhar, R. S. Rajmani, Amit Singh, Kithiganahalli Narayanaswamy Balaji

**Affiliations:** ^1^Department of Microbiology and Cell Biology, Indian Institute of Science, Bangalore, India; ^2^Department of Microbiology and Cell Biology, Centre for Infectious Disease Research, Indian Institute of Science, Bangalore, India

**Keywords:** mycobacteria, c-Abl, bone morphogenesis protein signaling, miRNA, TLR3, WhiB3, DosR

## Abstract

Mycobacteria propelled modulation of host responses is of considerable interest in the face of emerging drug resistance. Although it is known that Abl tyrosine kinases affect entry and persistence of mycobacteria, mechanisms that couple c-Abl to proximal signaling pathways during immunity are poorly understood. Loss-of-function of c-Abl through Imatinib, in a mouse model of tuberculosis or RNA interference, identified bone morphogenesis protein (BMP) signaling as its cellular target. We demonstrate that c-Abl promotes mycobacterial survival through epigenetic modification brought about by KAT5-TWIST1 at *Bmp* loci. c-Abl-BMP signaling deregulated iNOS, aggravating the inflammatory balance. Interestingly, BMP signaling was observed to have far-reaching effects on host immunity, as it attenuated TLR3 pathway by engaging miR27a. Significantly, these events were largely mediated *via* WhiB3 and DosR/S/T but not SecA signaling pathway of mycobacteria. Our findings suggest molecular mechanisms of host pathways hijacked by mycobacteria and expand our understanding of c-Abl inhibitors in potentiating innate immune responses.

## Introduction

*Mycobacterium tuberculosis* (Mtb), the causative agent of tuberculosis (TB), has garnered our attention as a leading cause of public health emergency due to its unprecedented toll on the world’s population. An estimated 1.4 million people succumbed to TB in 2015 and more importantly, an additional 1,00,000 rifampicin-resistant TB instances emerged (1). These statistics signal the pressing need to develop effective chemotherapy, which is able to meet the manifold objectives of TB control. Targeting of host factors critical to infection such as surface receptors, ion channels, kinases, phosphatases offers several benefits over conventional antibiotic therapy (2–4). Thus, Host-Directed Therapeutics could shorten the drawn-out course of TB treatment, economize combination drug therapy, minimize collateral damage, and even help retain lung function among TB patients (5).

Tyrosine kinase c-Abl, is activated in murine bone marrow derived macrophages infected with mycobacteria and was elucidated to mediate TNF-dependent apoptosis (6). This kinase was also shown to promote entry and enhance microbial survival through its inhibition of acidification of the Mtb-containing phagosome (7–10). In various other contexts, c-Abl is known to affect important cellular phenomena such as cytoskeletal dynamics (11), DNA damage responses (12), as well as autophagy (13). Thus, Mtb would benefit enormously by re-directing this central player of host immunity, leading us to question the limited exploration of c-Abl-mediated regulation during mycobacterial infection. Further, c-Abl is known to regulate a member of the TGF-β superfamily, the bone morphogenesis protein (BMP) pathway in osteoblasts (14, 15). In line with this literature, Andreu et al. report an enrichment of signaling pathways associated with bone tissue in macrophages infected with Mtb (16). Further, Das and colleagues suggest dormant mycobacteria persist in progenitors of bone cells (17). In addition, increasing evidences suggest an intersection of osteomyelitis with TB (18, 19). We therefore surmised the probable activation of BMP pathway during mycobacterial infection.

Importantly, apart from its role in bone metabolism, infection-induced BMP signaling has been poorly explored. BMP signaling was activated in human gastric mucosa afflicted with *Helicobacter pylori* contributing to increased apoptosis (20) and in a model of murine reovirus elicited encephalitis, where it was hypothesized to play an immune protective role (21). However, such literature is scarce and the roles of BMP signaling in effecting innate immune responses need further investigation. Briefly, during the activation of BMP signaling, BMP ligands bind to type I and II receptors and aid their association. This ensues the phosphorylation of type I receptors by the constitutively active (CA) kinase domains of type II receptors and drives the Smad signaling pathway through phosphorylation of receptor regulated Smad1/5/8. An association of these with Smad4 (co-Smad) results in a heteromeric complex that translocates to the nucleus and stimulates the expression of a wide range of target genes (22).

Mycobacteria are confronted with taxing intracellular milieu of host macrophages comprising toxic gasotransmittors such as nitric oxide (NO), carbon monoxide (CO) in addition to hypoxia. Mtb transcription factors WhiB3 and the DosR/S/T regulon are prime examples of virulence propagators responding to fluxes in these host-generated diatomic gases. WhiB3 has been implicated in its global regulation of virulence associated genes including the Esx-1 secretion pathway as well as complex immune-modulatory polyketides that subvert phagosomal maturation during infection (23). TF DosR has been of interest as mutants in DosR presented early transcriptional signatures of T-cell recruitment, activation, and increased T cell proliferation, signaling their failure to persist and cause disease (24). We utilized a panel of these Mtb mutants and questioned their relevance in the activation of c-Abl-dependent BMP signaling. Coincidently, Cumming et al. report BMPR1a, BMPR2, and Smad5 as WhiB3 targets (25) in RAW264.7 macrophages infected with *MtbΔWhiB3*.

Here, we decipher an intricate signaling mechanism linking tyrosine kinase c-Abl, chromatin modifier, KAT5 (lysine acetyl transferase), and transcription factor, TWIST1 acting at BMP2 and BMP4 promoters. Utilizing Imatinib as a tool, this molecular circuitry was observed to affect mycobacterial survival *via* a regulation of inducible nitric oxide synthase (iNOS). We found that NO, hypoxia, and CO responsive mycobacterial WhiB3 and DosR, but not the sec-dependent protein secretion pathway, orchestrate these phenomena. We also demonstrate key evidences of a cross-regulation among the TLR2 and TLR3 signaling cascades effected by BMP-dependent miR27a, which target TLR3 adaptor TICAM1 (TIR domain-containing adaptor molecule 1). Altogether, we reveal the contribution of Mtb effectors in impeding host protective responses.

## Materials and Methods

### Cells, Mice, and Bacteria

Primary macrophages were isolated largely from peritoneal exudates of BALB/c mice. Other mice used included C3H/HeJ (*Tlr4* mutant), C57BL/6J, and *tlr2*-knockout mice maintained in the Central Animal Facility, Indian Institute of Science (IISc). Both male and female mice were used in this study. Briefly, mice were injected immunoprecipitation with 1 ml of 8% Brewer thioglycollate. Mice were sacrificed after 4 days and peritoneal cells were harvested by lavage from peritoneal cavity with ice-cold PBS. The cells were cultured in DMEM (Gibco-Invitrogen/Thermo Fisher Scientific) containing 10% FBS (Gibco-Invitrogen/Thermo Fisher Scientific) for 24 h and adherent cells were used as peritoneal macrophages. Murine RAW264.7 macrophage cell line was obtained from an already-existing collection of Cell Repository at the National Center for Cell Sciences (NCCS), Pune, India. NIH3T3 and K562 cell lines were kind gifts from Prof. Anjali Karande (Dept. of Biochemistry, IISc) and Prof. Satees Raghavan (Dept. of Biochemistry, IISc). *M. tuberculosis* H37Rv Δ*dosR* was a kind gift from Dr. David Sherman, University of Washington, *M. tuberculosis* H37Rv Δ*secA* was a kind gift from Dr. William Jacobs, Albert Einstein College Medicine, *M. tuberculosis* H37Rv Δ*whiB3* and *M. tuberculosis* H37Rv Δ*whiB3*-comp were kind gifts from Dr. Amit Singh, IISc (61). All studies involving virulent mycobacterial strains were carried out at the BSL-3 facility at Centre for Infectious Disease Research (CIDR), IISc. Bacteria were grown to mid-log phase and used at 10 multiplicity of infection in all experiments unless mentioned otherwise.

### Aerosol Infection of Mice and Inhibitor Treatment

*Mycobacterium tuberculosis* H37Rv was grown in Middlebrook 7H9 medium (Difco) containing 0.2% glycerol, 0.05% Tween 80, and 10% ADC. Cultures were grown at 37°C to log phase. Culture was washed with PBS and passed 10 times each, through 26, 29, and 31 gauge needles to make single-cell suspensions of Mtb H37Rv. BALB/c mice were infected with Mtb using a Madison chamber aerosol generation instrument calibrated to 100 CFU/mice and maintained in securely commissioned BSL3 facility. 28 days postinfection, mice were given 10 doses of Imatinib (66 mg/kg) (Calbiochem, 504595) immunoprecipitation (every 2 days) (*n* = 10). On day 56, mice were sacrificed, left lobe of the lung and whole spleen was homogenized in sterile PBS, serially diluted and plated on 7H11 agar containing OADC to quantify Mtb H37Rv CFU. Superior lung lobe was fixed in formalin, embedded in paraffin and stained with hematoxylin and eosin to assess pathology. Granuloma features were characterized and assigned different scores: with necrosis (Score = 5), without necrosis (Score = 2.5), with fibrosis (Score = 1). Total granuloma scores were calculated by multiplying the characterized feature score with the number of granuloma in each lung.

### Antibodies

The following antibodies were utilized in this study: Cell Signaling Technology: BMPR2 (6979), p-Smad1/5/8 (9511), ID2 (3431), c-Abl (2862), KAT5 (12058), Flag (2368), pSTAT1 (9171), pSTAT3 (9131), and pSmad2/3 (8828). PeproTech Inc.: BMP2 (500-P195). Santa Cruz Biotechnology Inc.: BMP4 (sc-12721) and p-Tyrosine (PY99). Calbiochem: PCNA (NA-03). Abcam: TLR3 (ab62566), TICAM1 (ab13810), BMPR1a (ab38560), pPDGFR (ab5443), and p-c-KIT (ab5616). Sigma: iNOS (N7782) and β-ACTIN (A3854). Jackson Immuno Research: Goat anti-Rabbit HRP (111-035-045) and Mouse anti-Rabbit Light Chain specific HRP (211-032-171), DyLight 488 IgG Fraction Monoclonal Mouse Anti-Rabbit. Invitrogen: p-Threonine (PT-5H5). Tonbo Biosciences: PE Anti-Mouse F4/80 Antigen (BM8.1) (50-4801), APC Anti-Mouse Ly-6G (1A8) (20-1276). BD Biosciences: PE Anti-Mouse CD11c (HL3) (557401).

### Transient Transfection Studies

Transient transfection of 0.5 × 10^6^ RAW264.7 macrophages with 5 μg of mentioned plasmid constructs was performed using polyethylenimine (PEI, Sigma-Aldrich). In case of experiments involving siRNA, RAW264.7 macrophages were transfected with carefully titrated concentration of 100 nM siRNA. *Abl1, Twist1*, non-targeting siRNA, and siGLO Lamin A/C were obtained from Dharmacon as siGENOME SMARTpool reagents, which contain a pool of four different double-stranded RNA oligonucleotides. Transfection efficiency was found to be 70% in all the experiments as determined by the number of siGLO Lamin A/C positive cells in a microscopic field using fluorescent microscope. Further, 36 h post-transfection, the cells were treated or infected as indicated and processed for analysis.

### Luciferase Assay

0.5 × 10^6^ RAW264.7 cells were transfected with specific promoter luciferase plasmids using PEI (Sigma-Aldrich). After 36 h of transfection, cells were infected for 12 h where indicated. Cells were harvested and lysed in reporter lysis buffer (Promega, USA) and luciferase activity was assayed using luciferase assay reagent (Promega, USA). The results were normalized for transfection efficiencies by assay of β-galactosidase activity. *O*-nitrophenol β-d-galactopyranoside (ONPG, Hi-Media, RM582) was utilized for the assay.

### Treatment with Pharmacological Reagents

In all *in vitro* experiments, cells were treated with the given reagents 1 h prior to experimental treatments at the following concentrations: Imatinib (10 μM) (Gleevec), Alk5 inhibitor (10 nM) (Sigma, SB431542), Compound C (5 μM) (Calbiochem, 171260), Poly I:C (10 μg/ml) (Invivogen), Dibenziodolium chloride (10 μM) (DPI, Calbiochem, 300260), 1400W (Calbiochem, 100050), and IFN-γ (200 U/ml) (Biolegend). 2′,7′-Dichlorofluorescin diacetate (DCFDA, D6883) was purchased from Sigma. Recombinant PDGF-AB (100-00AB) and SCF (300-07) were purchased from PeproTech Inc. and used at concentrations of 10 and 100 ng/ml, respectively.

### Immunohistochemistry (IHC)

Microtome sections (5 μm) were obtained from 3.6% formaldehyde-fixed, decalcified, and paraffin-embedded tissues using Leica RM2245 microtome (Leica). These sections were first deparaffinized, subjected to antigen retrieval by boiling in 10 mM citrate buffer (pH 6.0) for 10 min, treated with 1% H_2_O_2_ for 10 min, and blocked with 5% BSA (Sigma-Aldrich) for 1 h at room temperature. The tissue sections were further incubated with primary antibodies overnight. After incubation with anti-rabbit HRP-conjugated secondary antibody for 90 min, sections were stained with 0.05% diaminobenzidine (DAB, Sigma-Aldrich) in 0.03% H_2_O_2_ solution and counterstained with hematoxylin, dehydrated, and mounted. Stained tissue sections were imaged with Axio Scope A1 microscope (Zeiss) at indicated magnification. All experiments were performed with appropriate isotype-matched control antibodies and a minimum of three tissue sections were assessed.

### Cryosection Preparation

The murine lung was rapidly frozen in liquid nitrogen in optimal cutting temperature (OCT) media (Jung, Leica). Cryosections of 10–15 μm were taken in Leica CM 1510 S or Leica CM 3050 S cryostat with the tissue embedded in OCT onto the glass slides and stored at −80°C.

### Immunofluorescence

Cryosections were thawed to room temperature and after blocking with 2% BSA containing saponin, the sections were stained for specific antibodies at 4°C overnight. The sections were incubated with DyLight 488-conjugated secondary antibody for 2 h and nuclei stained with DAPI. A coverslip was mounted on the section with glycerol as the medium. Confocal images were taken on Zeiss LSM 710 Meta confocal laser scanning microscope (Carl Zeiss AG) using a plan-Apochromat 40×/1.4 Oil DIC objective (Carl Zeiss AG) and images were analyzed using ZEN 2009 software.

### Quantification of miRNA Expression

For detection of miR27a, total RNA was isolated from the indicated cells/tissues using the TRI reagent. Quantitative real-time RT-PCR for miR27a (assay ID 00408) was performed using TaqMan miRNA assays (Life Technologies, 4324018) as per manufacturer’s instructions. U6 snRNA (assay ID 4427975) was used for normalization.

### Chromatin Immunoprecipitation (ChIP) Assay

Chromatin immunoprecipitation assays were performed using a protocol provided by Upstate Biotechnology, USA, with certain modifications. Macrophages were fixed with 1.42% formaldehyde for 7 min at room temperature followed by inactivation of formaldehyde with addition of 125 mM glycine. Cells were lysed using 0.1%SDS lysis buffer (with 1% Triton X-100) and chromatin was sheared in Bioruptor (Diagenode) Sonicator (High Power, 70 cycles of 30 s pulse ON and 45 s pulse OFF). Chromatin extracts containing DNA fragments with an average size of 500 bp were immunoprecipitated using specific antibody or isotype IgG. Immunoprecipitated complexes were sequentially washed with Wash Buffer A [50 mM Tris–HCl (pH 8.0), 500 mM NaCl, 1 mM EDTA, 1% Triton X-100, 0.1% Sodium deoxycholate, 0.1% SDS, and protease/phosphatase inhibitors], Wash Buffer B [50 mM Tris–HCl (pH 8.0), 1 mM EDTA, 250 mM LiCl, 0.5% NP-40, 0.5% Sodium deoxycholate and protease/phosphatase inhibitors], TE buffer [10 mM Tris–HCl (pH 8.0), 1 mM EDTA], and eluted in elution buffer [1% SDS, 0.1 M NaHCO_3_]. After treating the eluted samples with RNase A and Proteinase K, DNA was precipitated using phenol–chloroform–ethanol method. Purified DNA was analyzed by quantitative real-time RT-PCR. All values in the test samples were normalized to amplification of the specific gene in Input and IgG pull down and represented as fold change. All ChIP experiments were repeated at least three times and the primers utilized are listed in Table 1.

**Table 1:** Primers used in this study.

No.	Gene	Forward primer (5′–3′)	Reverse primer (5′–3′)
1.	*Bmpr2*	ttgggataggtgagagtcgaat	tgtttcacaagattgatgtcccc
2.	*Bmpr1a*	aacagcgatgaatgtcttcgag	gtctggaggctggattatggg
3.	*Bmp2*	gggacccgctgtcttctagt	tcaactcaaattcgctgaggac
4.	*Bmp4*	ttcctggtaaccgaatgctga	cctgaatctcggcgacttttt
5.	*Bmp7*	acggacagggcttctcctac	atggtggtatcgagggtggaa
6.	*Id2*	atgaaagccttcagtccggtg	agcagactcatcgggtcgt
7.	*Gapdh*	gagccaaacgggtcatcatct	gaggggccatccacagtctt
8.	*Twist1*	ggacaagctgagcaagattca	cggagaaggcgtagctgag
9.	*Ticam1*	aacctccacatcccctgtttt	gccctggcatggataacca
10.	*Ifn-α*	tctgatgcagcaggtggg	agggctctccagacttctgctctg
11.	*Ifn-β*	cagctccaagaaaggacgaac	ggcagtgtaactcttctgcat

**For chromatin immunoprecipitation assays**			
1.	*Bmp2*	ccctctgtgggttgctaatccg	gagaaagaccagaagcaggggc
2.	*Bmp4*	gcccgggtccggaagatt	gggaagcccagactcccatg

**For 3′UTR cloning**			
1.	*Ticam1* *3′UTR*	cgagctcgggctagagtgacaagattggac	gctctagagcacaggaccctccctatgt

**For site-directed mutagenesis of 3′UTR**			
1.	*Ticam1* Δ*miR27a*	gtaaacttcattcacgatatcacatgctgttcatag	ctatgaacagcatgtgatatcgtgaatgaagtttac

### Nitrite Estimation

Cells were cultured in a 96-well plate, using triplicates for each condition and proper controls. Cell supernatants were collected and 50 μl was transferred to a new 96-well plate. Simultaneously, standard stock solution was diluted to obtain a standard curve. 50 μl of Sulfanilamide Solution was added to each sample, control well, and mixed well. It was incubated at room temperature for 10 min in the dark. Further, 50 μl of *N*-1-napthylethylenediamine dihydrochloride solution was added to each sample, control well, and mixed well. The plate was incubated at room temperature for 10 min in the dark. Absorbance was measured immediately at 540 nm.

### Estimation of Reactive Oxygen Species (ROS) Using DCFDA

Treated cells were loaded with the DCFDA dye (at a final concentration of 20 μM) and kept for 30 min in the dark, in a conventional incubator (37°C, 5% CO_2_). Carboxy-H2DCFDA containing medium was discarded and cells were washed twice with PBS. ROS was assessed immediately by analyzing cells utilizing fluorescence plate reader at 529 nm.

### Immunoblotting

Cells were washed with ice-cold PBS, scraped from the culture dish and collected by centrifugation. Cell pellets were lysed in RIPA buffer (50 mM Tris–HCl, pH 7.4, 1% NP-40, 0.25% Sodium deoxycholate, 150 mM NaCl, 1 mM EDTA, 1 mM PMSF, 1 mg/ml of each aprotinin, leupeptin, pepstatin, 1 mM Na_3_VO_4_, 1 mM NaF). Whole cell lysate was collected by centrifuging lysed cells at 13,226 × *g*, 15 min at 4°C. Bradford’s method of protein estimation was used to assess protein concentration in each cell lysate. Equal protein from each sample was subjected to SDS-PAGE and subsequently transferred onto polyvinylidene difluoride membranes (Millipore, USA) by the semidry (Bio-Rad, USA) method. Membranes were blocked with 5% non-fat dry milk powder (bovine, Sigma-Aldrich) in TBST [20 mM Tris–HCl (pH 7.4), 137 mM NaCl, and 0.1% Tween 20] for 60 min. The blots were incubated overnight at 4°C with primary antibodies diluted in TBST with 5% BSA. After washing with TBST, blots were incubated with necessary secondary antibody conjugated to HRP (Jackson Immunoresearch, USA) diluted in 5% non-fat milk for 4 h. After further washes in TBST, the immunoblots were developed with ECL reagent (Perkin Elmer, USA). β-ACTIN was used as loading control.

### Nuclear and Cytosolic Fractionation

Cells were treated as indicated, harvested by centrifugation, and gently resuspended in ice-cold Buffer A (10 mM HEPES pH 7.9, 10 mM KCl, 0.1 mM EDTA, 0.1 mM EGTA, 1 mM DTT, and 0.5 mM PMSF). After incubation on ice for 15 min, cell membranes were disrupted with 10% NP-40 and the nuclear pellets were recovered by centrifugation at 13,226 × *g* for 15 min at 4°C. The supernatants from this step were used as cytosolic extracts. Nuclear pellets were lysed with ice-cold Buffer C (20 mM HEPES pH7.9, 0.4 M NaCl, 1 mM EDTA, 1 mM EGTA, 1 mM DTT, and 1 mM PMSF) and nuclear extracts were collected after centrifugation at 13,226 × *g* for 20 min at 4°C.

### Immunoprecipitation Assay

Immunoprecipitation (IP) assays were performed using a protocol provided by Millipore, USA, with certain modifications. Briefly, macrophages were gently lysed in ice-cold RIPA buffer on an orbital shaker. Whole cell lysate was collected by centrifuging lysed cells at 13,226 × *g*, 10 min at 4°C. Cell lysate was pre-cleared with Protein A (Bangalore Genei, 62211018005A) beads blocked with 2% BSA. Bradford’s method of protein estimation was used to assess protein concentration in each sample. Cell lysates containing equal protein were incubated with respective antibody or IgG at 4°C for 6 h on the orbital shaker. The immunocomplexes were captured using Protein A agarose at 4°C for 4 h. The beads were harvested, washed (with ice-cold RIPA buffer) and boiled in 5× Laemmli buffer for 10 min. The samples were separated by SDS-PAGE and further subjected for immunoblotting.

### Assessment of Poly I:C Induced Responses

BALB/c mice (*n* = 16) were infected with mid-log phage Mtb H37Rv, as mentioned above. On day 56, mice (*n* = 8) were injected with 10 mg/kg Poly I:C (intravenously), 12 h prior to sacrifice. RNA was isolated from the lungs and spleen of these mice and assessed for TLR3 stimulated signaling. PBS (*n* = 3), Poly I:C (*n* = 8) injected and Mtb H37Rv (*n* = 8) infected mice were used as controls.

### *Ticam1* 3′UTR Wild-Type (WT) and Mutant Generation

The full length 3′UTR of *Ticam1* was PCR amplified and cloned into a pmirGLO vector using the restriction enzyme pair SacI and XbaI. The miR27a binding site in the 3′UTR of *Ticam1*, was mutated by nucleotide replacements through site-directed mutagenesis using the inverse PCR method. The forward and reverse primers were perfectly complementary and comprised the desired mutations flanked by 10–15 nucleotides on each side (Table 1). The newly synthesized plasmid was separated from the parental plasmid by digesting the reaction with the restriction enzyme DpnI followed by transformation and screening for mutants.

### Quantitative Real-time RT-PCR

Macrophages were treated or infected as indicated and total RNA was isolated using TRI reagent (T9424, Sigma-Aldrich, USA). For RT-PCR, 1 μg of total RNA was converted into cDNA using First Strand cDNA synthesis kit (M3682, Promega). Quantitative real-time RT-PCR was performed with SYBR Green PCR mixture (F416, Thermo Fisher Scientific). All the experiments were repeated at least three times, independently, to ensure reproducibility of the results. *Gapdh* was used as internal control. The primers used for quantitative real-time RT-PCR are summarized in Table 1.

### Statistical Analysis

Levels of significance for comparison between samples were determined by the Student’s *t*-test distribution or one-way ANOVA followed by Tukey’s multiple comparisons. The data in the graphs are expressed as the mean ± SE for the values from at least three or more independent experiments and *P* values < 0.05 were defined as significant. GraphPad Prism 5.0 software (GraphPad Software) was used for all the statistical analysis.

## Results

### c-Abl Co-Ordinates Mycobacteria-Stimulated BMP Signaling in Host Macrophages upon Infection

**Figure 1:**
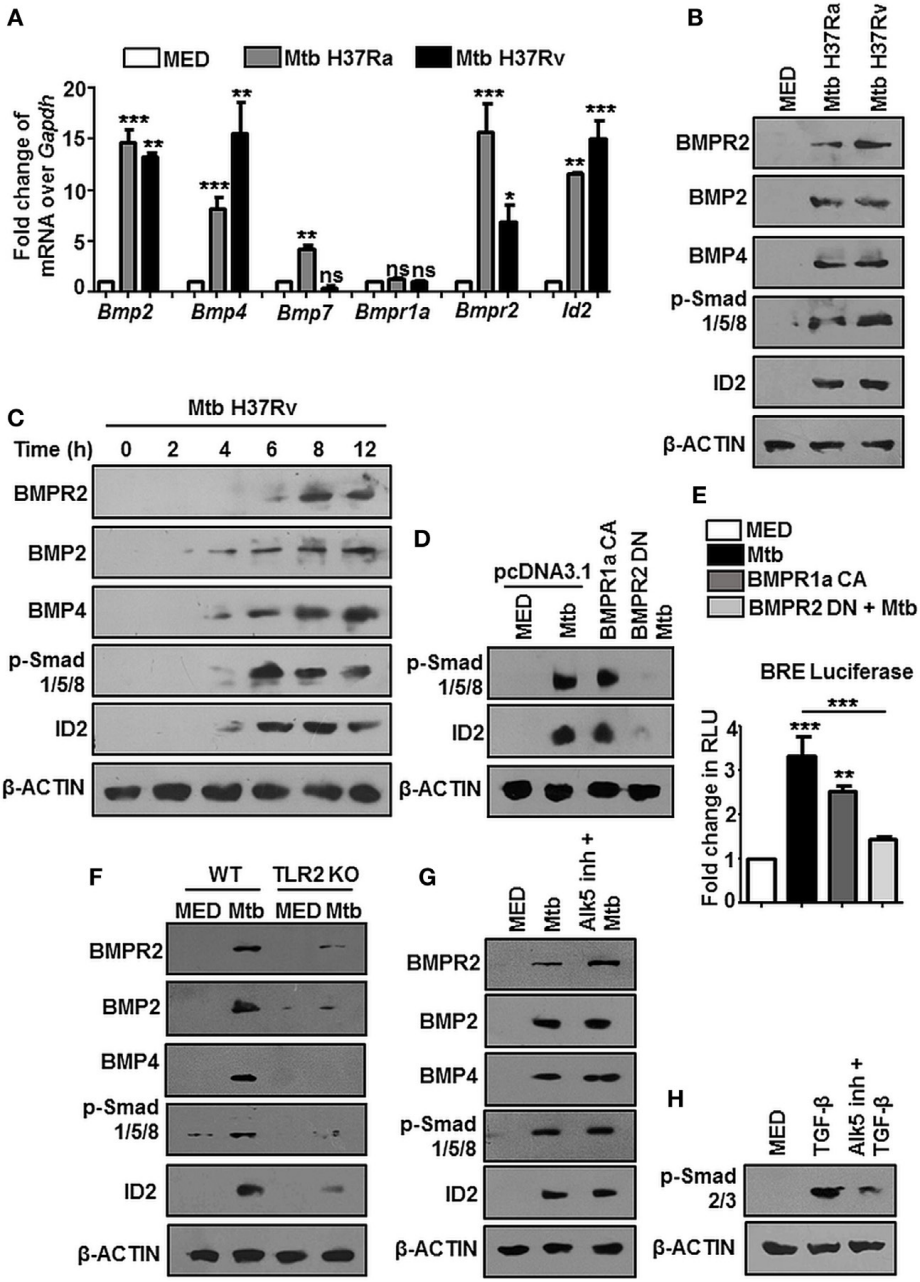
Mycobacteria activate TLR2-dependent bone morphogenesis protein (BMP) signaling upon infection. Mouse peritoneal macrophages were infected with *Mycobacterium tuberculosis* (Mtb) H37Ra and Mtb H37Rv (multiplicity of infection 1:10) for 12 h. Transcript analysis of BMP signaling activation markers was performed by RT-PCR **(A)**. Immunoblotting was performed to assess total protein levels of BMPR2, BMP2, BMP4, p-Smad1/5/8, and ID2 **(B)**. Time kinetic analysis of hallmarks of BMP signaling activation upon infection with Mtb H37Rv was assessed by immunoblotting **(C)**. RAW264.7 macrophages were transiently transfected with pcDNA3.1/BMPR1a constitutively active (CA) construct/BMPR2 dominant-negative (DN) construct and infected with Mtb where indicated. Protein levels of targets of BMP signaling were assessed **(D)** and BMP responsive element (BRE) activity was assessed by luciferase assay **(E)**. Activation of BMP signaling was assessed in peritoneal macrophages of WT and TLR2 KO mice **(F)**. Activation of BMP signaling was assessed by immunoblotting in peritoneal macrophages pretreated (1 h) with TGF-β type-I receptor inhibitor, Alk5 **(G)**. Validation of Alk5 inhibitor. Murine peritoneal macrophages were pretreated with Alk5 inhibitor (1 h), followed by TGF-β treatment (2 h). TGF-β responsive p-Smad2/3 was assessed by immunoblotting **(H)**. All *in vitro* Mtb infections were performed for 12 h. Data represent the mean ± SEM for five values from three independent experiments. WT, wildtype; KO, knockout; ns, not significant, *P < 0.05, **P < 0.005, ***P < 0.0001 (one-way ANOVA followed by Tukey’s multiple-comparisons test). All blots are representative of three independent experiments.

At the outset, we asked if BMP signaling is mycobacteria responsive. We examined the expression of hallmarks of BMP signaling during mycobacterial infection and found transcript levels of *Bmp2, Bmp4, Bmpr2*, and *Id2* were consistently upregulated upon infection of mouse peritoneal macrophages with Mtb H37Ra as well as virulent Mtb H37Rv (Figure 1A). There was no change in the transcript levels of BMPR1a, emphasizing that phosphorylation of BMPR1a suffices the activation of BMP signaling. BMPR2, BMP2, BMP4, p-Smad1/5/8, and ID2 were also assessed at the protein level (Figure 1B). No significant differences were observed among the transcript or protein abundances of Mtb H37Ra and Mtb H37Rv-infected macrophages with respect to BMP signaling (Figure S1A in Supplementary Material). Further, it was found that activated BMP signaling was a delayed event (Figure 1C; Figure S1B in Supplementary Material). We established that mycobacteria-activated BMP signaling required canonical receptors BMPR2 and BMPR1a, as overexpressed CA BMPR1a spurred an activation of BMP signaling, while a dominant-negative form of BMPR2 failed to activate the cascade even during infection (Figure 1D). The same was also assessed utilizing a BMP responsive element (BRE) tagged to luciferase (Figure 1E). Among pattern recognition receptors (PRRs), TLR2 is known to play important roles in arbitrating the outcome of mycobacterial infections (26). TLR2 was observed to be indispensable for the ensuing activation of BMP signaling (Figure 1F). In order to assess the possibility of TGF-β receptors regulating BMP signaling, we evaluated the activation of BMP and TGF-β cognate response regulators p-Smad2/3 in the presence of ALK5 (a Type I TGF-β receptor kinase) inhibitor and found that BMP signaling operates independent of the TGF-β clade (Figures 1G,H). Thus, we established TLR2-dependent activation of BMP signaling during Mtb infection.

**Figure 2:**
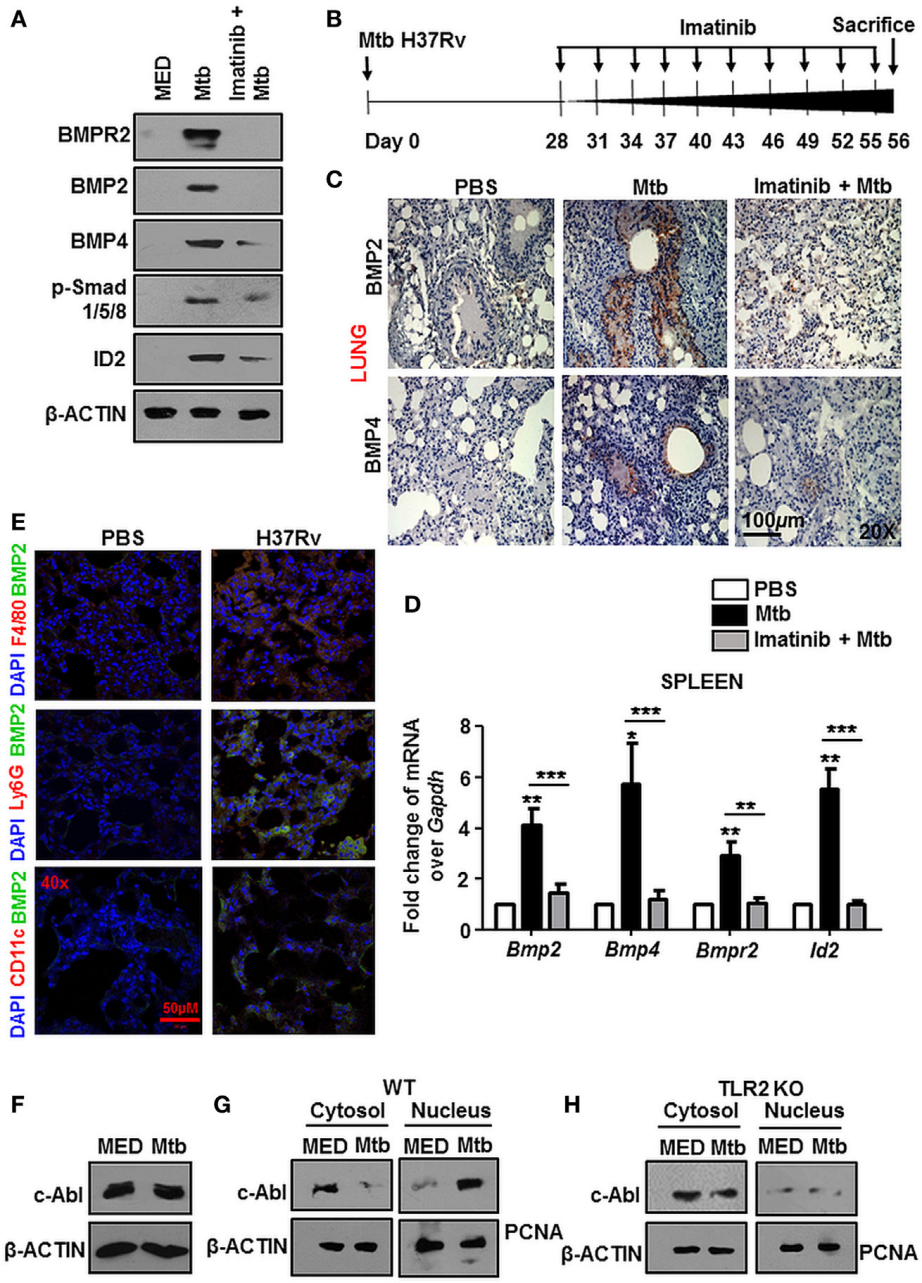
Nuclear localized c-Abl drives mycobacteria-activated bone morphogenesis protein (BMP) signaling. Mouse peritoneal macrophages were pretreated for 1 h with Imatinib, followed by *Mycobacterium tuberculosis* (Mtb) infection and assessed for the activation of BMP signaling by immunoblotting **(A)**. Model of established tuberculosis: BALB/c mice were infected with 100 CFU of Mtb (aerosol) and the infection was allowed to establish for 28 days. Subsequently, c-Abl inhibitor, Imatinib (66 mg/kg, intraperitoneal) was administered to the mice every 2 days for the next 28 days. Assessment was performed at the end of 56 days. Schematic of the *in vivo* experiment **(B)**. IHC was performed on lung sections of these mice stained for BMP2 and BMP4 **(C)** and transcript analysis of BMP signaling activation markers was performed by RT-PCR in their spleens **(D)** (*n* = 4, each group). Representative immunofluorescence images of lung cryosections of mice (as explained in Figure 2B) stained for macrophages (F4/80) or neutrophils (Ly6G) or dendritic cells (CD11c) with BMP2 **(E)**. Total protein levels of c-Abl **(F)**, and its nuclear-cytosolic fractionation in WT **(G)**, TLR2 KO **(H)** murine peritoneal macrophages were assessed by immunoblotting post Mtb infection. All in *vitro* Mtb infections were performed for 12h. All data represent the mean ± SEM for five values from three independent experiments. WT, wildtype; KO, knockout; IHC, immunohistochemistry; ns, not significant, **P < 0.005, ***P < 0.0001 (one-way ANOVA followed by Tukey’s multiple-comparisons test). All blots are representative of three independent experiments. Magnifications and scale are represented on the indicated images.

Earlier evidences suggested that c-Abl tunes BMP signaling through the direct phosphorylation of the BMP receptor BMPR1a in osteoblasts and thus activates downstream events (14, 15). In this respect, we found that c-Abl interacted poorly with BMPR1a during mycobacterial infection (Figure S1C in Supplementary Material). Further, although its threonine phosphorylation was robustly induced upon Mtb infection, its tyrosine phosphorylation status remained relatively unchanged (Figure S1D in Supplementary Material). These findings excluded a role for c-Abl in activating BMP signaling through its phosphorylation of BMPR1a. We next utilized Imatinib, a small-molecule inhibitor targeted against c-Abl, to negate the role of c-Abl in activating mycobacteria-dependent BMP signaling. Yet, when assessed *in vitro* at the protein level (Figure 2A), remarkably, Imatinib treatment curbed Mtb activated BMP signaling.

In addition to inhibiting Abl tyrosine kinases, Imatinib targets two other receptor tyrosine kinases, namely, c-KIT and platelet-derived growth factor receptor (PDGFR) with high affinity, albeit to varying degrees. Consistent reports suggest that the 50% inhibitory concentration (IC_50_) values of Imatinib for c-Abl are approximately 10-fold lower than those for PDGFR and c-KIT (27–29), suggesting that Abl kinases are the foremost targets of Imatinib. In this context, we demonstrated a titration of Imatinib concentrations and assessed the phosphorylation of c-Abl, PDGFR and c-KIT in macrophages, NIH3T3, and K562 cells. We found that Imatinib demonstrated a substantial inhibition of c-Abl at a concentration of 5 μM and significantly reduced c-Abl phosphorylation at 10 μM (Figure S1E in Supplementary Material), while PDGF (Figure S1F in Supplementary Material) and c-Kit (Figure S1G in Supplementary Material) phosphorylation was inhibited at higher concentrations of Imatinib.

In order to assess this *in vivo*, we generated a mouse model of established TB utilizing Mtb H37Rv. Groups of mice were infected with Mtb H37Rv for 28 days, followed by treatment with vehicle or Imatinib at 28 days postinfection, for a total duration of 56 days (Figure 2B). In line with the *in vitro* data, lung IHC (Figure 2C) and transcript levels of hallmarks of BMP signaling in spleen (Figure 2D) of Mtb-infected mice demonstrated abrogated levels upon Imatinib treatment. Immunofluorescence studies focusing on the cell types expressing BMP2 revealed that macrophages predominantly display activated BMP signaling (appearance of yellow coloration through the co-localization of green BMP2 and red F4/80 signal), as do CD11c^+^ cells (mostly dendritic cells) to a lesser extent. Ly6G^+^ (neutrophils) cells however, displayed a poor activation of BMP signaling upon mycobacterial infection (Figure 2E). c-Abl overexpression sufficed the activation of BMP signaling, while a kinase dead (KD) form of c-Abl failed to bring the effect, highlighting that kinase activity of c-Abl was essential for the initiation of Mtb-driven BMP signaling (Figure S2A in Supplementary Material). While cellular c-Abl levels were unaffected during mycobacterial infection (Figure 2F), we observed its redistribution in the cytosolic and nuclear compartments. Upon infection, c-Abl was found to translocate increasingly to the nucleus (Figure 2G). This event was TLR2 driven (Figure 2H) and independent of TLR4 (Figure S2B in Supplementary Material). Thus, c-Abl was observed to regulate BMP signaling in macrophages, as in osteoblasts, yet the events leading to such an activation were unclear in the present scenario.

### Chromatin Remodeling Results in Transcriptional Rewiring of *Bmp2/4* Locus Driving the Activation of BMP Signaling

**Figure 3:**
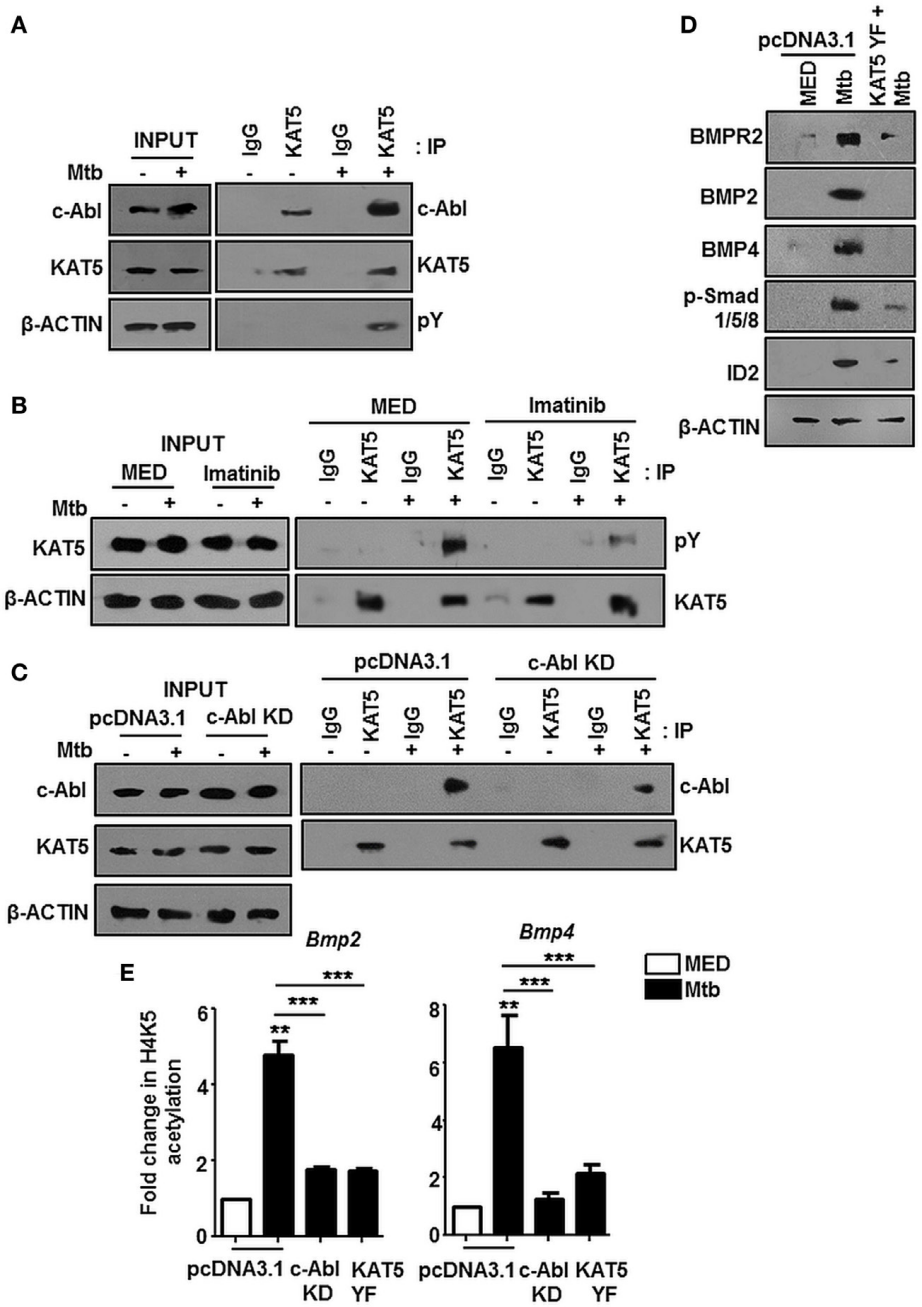
c-Abl recruited KAT5 mediates epigenetic activation of BMP2 and BMP4. KAT5 was immunoprecipitated in mouse peritoneal macrophages infected with *Mycobacterium tuberculosis* (Mtb) and immunoprecipitates were assessed for c-Abl and tyrosine phosphorylation of KAT5 (pY) by immunoblotting **(A)**. Imatinib pretreated (1 h) peritoneal macrophages were infected with Mtb as indicated. KAT5 was immunoprecipitated and immunoprecipitates were assessed for pY status of KAT5 by immunoblotting **(B)**. RAW264.7 macrophages were transiently transfected with pcDNA3.1/c-Abl KD construct followed by Mtb infection where indicated. KAT5 immunoprecipitation was performed in these lysates, and immunoprecipitates were assessed for c-Abl-KAT5 interaction by immunoblotting **(C)**. RAW264.7 macrophages were transiently transfected with pcDNA3.1 or KAT5YF (dominant-negative) construct followed by Mtb infection and assessed for the activation hallmarks of bone morphogenesis protein (BMP) signaling by immunoblotting **(D)**. RAW264.7 macrophages were transiently transfected with indicated constructs followed by Mtb infection where indicated. H4K5 acetylation was assessed at *Bmp2* and *Bmp4* promoters by chromatin immunoprecipitation **(E)**. All *in vitro* Mtb infections were performed for 12 h, and data represent the mean ± SEM for five values from three independent experiments. KD, kinase dead; **P < 0.005, ***P < 0.0001 (one-way ANOVA followed by Tukey’s multiple-comparisons test) and all blots are representative of three independent experiments.

**Figure 4:**
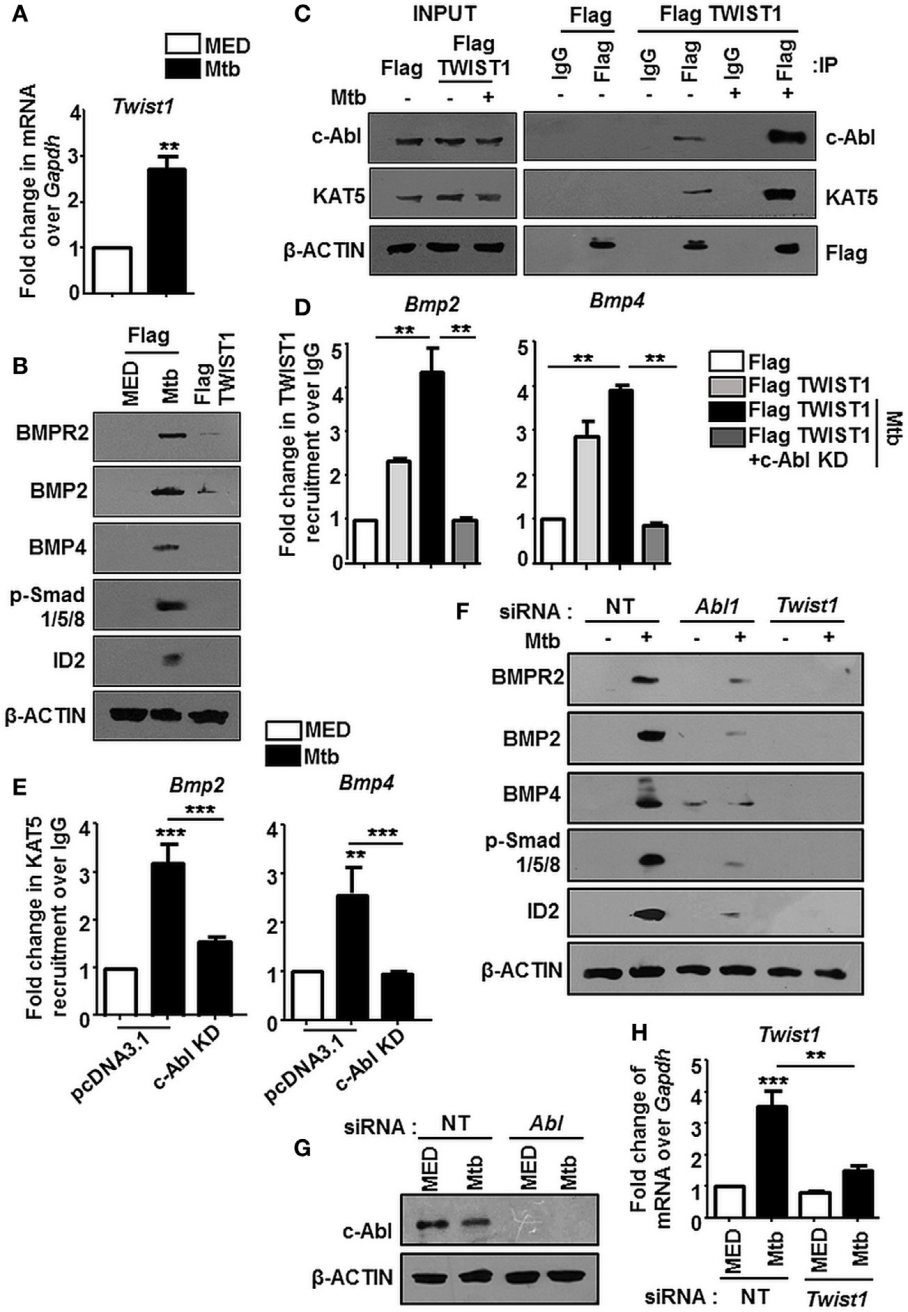
TWIST1 is essential for mycobacteria-orchestrated bone morphogenesis protein (BMP) signaling. Transcript analysis of *Twist1* upon *Mycobacterium tuberculosis* (Mtb) infection **(A)**. RAW264.7 macrophages were transiently transfected with Flag or Flag TWIST1 construct followed by Mtb infection as indicated and assessed for the activation of BMP signaling by immunoblotting **(B)**. RAW264.7 macrophages were transiently transfected with Flag or Flag TWIST1 constructs followed by Mtb infection. FLAG was immunoprecipitated and assessed for c-Abl and KAT5 by immunoblotting **(C)**. RAW264.7 macrophages were transiently transfected Flag TWIST1 construct or co-transfected with c-Abl KD construct where indicated, followed by Mtb infection. TWIST1 recruitment was assessed at BMP2 and BMP4 promoters through Flag immunoprecipitation and subsequent chromatin immunoprecipitation (ChIP) **(D)**. RAW264.7 macrophages were transiently transfected with pcDNA3.1/c-Abl KD construct and infected with Mtb as shown. KAT5 recruitment at TWIST1 binding region of *Bmp2* and *Bmp4* promoters was assessed by ChIP **(E)**. RAW264.7 macrophages were transiently transfected with *Abl1* siRNA/*Twist1* siRNA, infected with Mtb, and assessed for the activation of BMP signaling markers by immunoblotting **(F)**. Validation of *Abl1* siRNA **(G)** and *Twist1* siRNA **(H)** in RAW264.7 macrophages. All *in vitro* Mtb infections were performed for 12 h, and data represent the mean ± SEM for five values from three independent experiments. KD, kinase dead; **P < 0.005, ***P < 0.0001 (one-way ANOVA followed by Tukey’s multiple-comparisons test) and all blots are representative of three independent experiments.

The nuclear translocation of c-Abl during mycobacterial infection excluded a conventional role for c-Abl at the cytoplasmic membrane in phosphorylating receptor BMPR1a (Figure 2). c-Abl possesses a less examined DNA binding domain that was demonstrated to bind and recruit transcriptional factors such as p53 (30). We revisited the possibility of c-Abl regulating mycobacteria responsive BMP ligands, BMP2 and BMP4. A prediction of potential transcription factor binding sites using MatInspector (Genomatix) identified c-Abl binding sites in the 2 kb region of the promoters of BMP2 and BMP4 (Figure S2C in Supplementary Material). Further, a recent study demonstrated that lysine acetyl transferase KAT5, also known as Tip60, is a c-Abl partner (31). Here, Kaidi and Jackson reported c-Abl facilitated tyr44 phosphorylation of KAT5, thus rendering it active. We found an increased association of c-Abl with endogenous KAT5 during Mtb infection (Figure 3A). In addition, KAT5 levels were unperturbed during infection but its phosphorylated tyrosines (pY) were significantly increased. Further, inhibition of kinase activity of c-Abl brought about by Imatinib also resulted in a loss of phosphorylated tyrosine of KAT5 (Figure 3B) during infection. Interestingly, RAW264.7 macrophages transfected with c-Abl KD elucidate reduced c-Abl-KAT5 interaction (Figure 3C) during infection. When KAT5YF (Tyr44 was mutated to a non-phosphorylatable phenylalanine) was overexpressed in macrophages, followed by Mtb infection, BMP signaling failed to be activated (Figure 3D), thus underscoring the importance of c-Abl phosphorylated KAT5 in driving BMP signaling during Mtb infection. We observed increased H4K5 acetylation at the promoters of BMP2 and BMP4 upon Mtb infection, while overexpression of c-Abl KD or KAT5YF failed to bring about a similar effect (Figure 3E), suggesting an important role for KAT5-mediated H4K5 acetylation and subsequent activation of BMP2 and BMP4 promoters. Yet, c-Abl does not possess the ability for transcriptional activation. The promoters of BMP2/4 also indicated multiple TWIST1 binding sites (Figure S2C in Supplementary Material). TWIST1 has been demonstrated to play varied roles in the regulation of BMP signaling (32–34). We observed elevated levels of TWIST1 upon Mtb infection (Figure 4A). However, TWIST1 overexpression failed to simulate the activation of BMP signaling (Figure 4B), suggesting the necessity of mycobacteria triggered molecular machinery in orchestrating its activation. In support of these hypotheses, we observed an increased association of c-Abl-KAT5-TWIST1 during Mtb infection (Figure 4C). In line with these observations, we also confirmed the increased recruitment of TWIST1 to BMP2/4 promoters in the course of infection, which was hampered in the presence of c-Abl KD (Figure 4D). In addition, there was an increased recruitment of KAT5 to the promoters of BMP2 and BMP4 at their TWIST1 binding sites (Figure 4E), which was lost upon transfection of c-Abl KD in RAW264.7 macrophages. siRNA-mediated depletion of c-Abl or TWIST1 failed to trigger Mtb activated BMP signaling (Figures 4F–H), thus establishing a conclusive role for TWIST1 in conjunction with c-Abl-KAT5 in coordinating the regulation of BMP pathway.

### Pathogenic Mycobacteria Coopt Host c-Abl to Promote Their Survival

**Figure 5:**
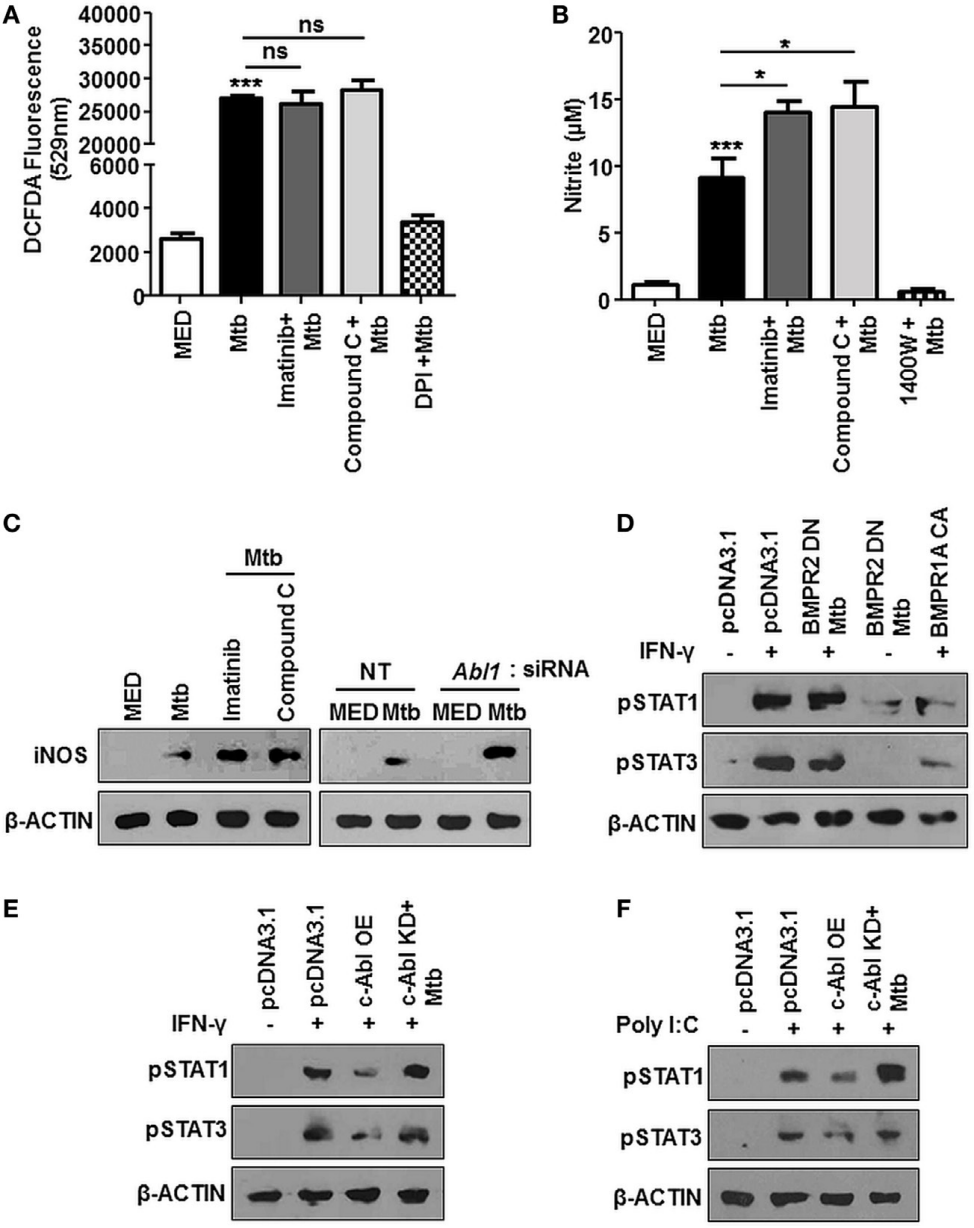
Bone morphogenesis protein (BMP) signaling regulates iNOS, aiding evasion of host immune system during mycobacterial infection. Mouse peritoneal macrophages were pretreated with Imatinib/Compound C/DPI/1400W followed by *Mycobacterium tuberculosis* (Mtb) infection where indicated. Reactive oxygen species production was estimated by dichlorofluorescin diacetate (DCFDA) fluorescence **(A)** and levels of nitrite were assessed by Griess assay **(B)**. iNOS was assessed by immunoblotting in mouse peritoneal macrophages pretreated with Imatinib or Compound C, followed by Mtb infection [**(C)**, left**]** and in RAW264.7 macrophages transiently transfected with *Abl1* siRNA subsequently followed by Mtb infection [**(C)**, right]. Phosphorylated STAT1 and STAT3 were assessed by immunoblotting in each of the following instances **(D–F)**. RAW264.7 macrophages were transiently transfected with pcDNA3.1 or BMPR2 DN or BMPR1a CA construct followed by pretreatment with IFN-γ (200 U/ml, 1 h) and subsequent Mtb infection as indicated **(D)**. RAW264.7 macrophages were transiently transfected with pcDNA3.1/c-Abl OE/c-Abl kinase dead (KD) construct followed by pre-treatment with IFN-γ (200 U/ml, 1 h) and subsequent Mtb infection as indicated **(E)**. RAW264.7 macrophages were transiently transfected with pcDNA3.1/c-Abl OE/c-Abl KD construct followed by pretreatment with Poly I:C (1 h) and subsequent Mtb infection as indicated **(F)**. All *in vitro* Mtb infections were performed for 12 h, and data represent the mean ± SEM for five values from three independent experiments. ns, not significant; *P < 0.05, **P < 0.005, ***P < 0.0001 (one-way ANOVA followed by Tukey’s multiple-comparisons test) and all blots are representative of three independent experiments.

We attempted to decipher the signaling events resulting in reduced survival of mycobacteria upon treatment with Imatinib. ROS and Reactive Nitrogen Intermediates (RNI) are recognized as important players mediating host protection during mycobacterial infection (35, 36). In this respect, we found Mtb stimulated ROS levels were unaffected in the presence of Imatinib or Compound C (Figure 5A), DPI treated cells were used as a control. Compound C (or Dorsomorphin) is an ALK2 (ActR1a), ALK3 (BMPR1a), and ALK6 (BMPR1B) inhibitor known to affect BMP-mediated Smad1/5/8 phosphorylation (37), while DPI (Diphenyleneiodonium) inhibits the production of ROS through the inhibition of NAD(P)H oxidase. However, cellular levels of nitrite were enhanced upon pretreatment of cells with both these inhibitors (Figure 5B), *N*-(3-(aminomethyl)benzyl)acetamidine (1400W) is a selective inhibitor for iNOS and was used as a control. This was also corroborated by elevated iNOS in the presence of Imatinib and Compound C (Figure 5C, left). Further, macrophages transfected with siRNA targeted against *Abl1* recapitulated increased iNOS levels (Figure 5C, right). A study of the regulators of iNOS revealed that IFN-γ stimulated STAT1 and STAT3 phosphorylation are abrogated in response to mycobacterial infection (38), although there exist contrary reports. We found that Mtb requires activated BMP signaling to prevent the phosphorylation of STAT1/3 (Figure 5D). Mycobacteria regulated abrogation of STAT1/3 phosphorylation was also dependent on c-Abl, as c-Abl overexpression exhibited similar effects (Figure 5E). Further, Poly I:C induced activation of STAT1/3 was also inhibited by transient overexpression of c-ABL WT, yet this effect was not observed when c-Abl KD was overexpressed (Figure 5F). Taken together, we propose that Mtb employ c-Abl activated BMP signaling to maintain a check on nitric oxide induced upon infection in murine macrophages.

**Figure 6:**
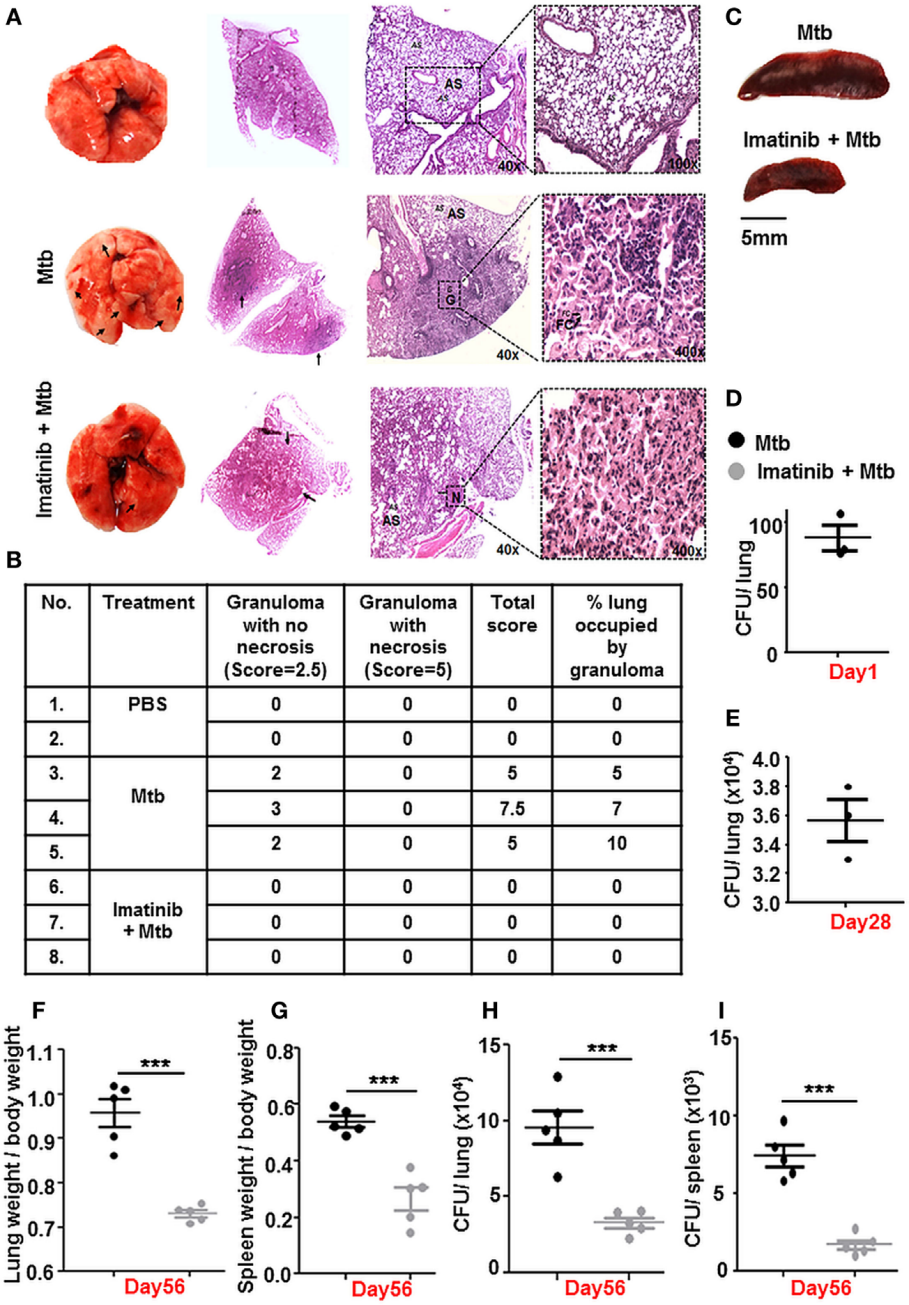
c-Abl regulates mycobacterial burden in a mouse model of established tuberculosis. Representative lung morphology and histopathology of mice injected with PBS, infected with *Mycobacterium tuberculosis* (Mtb) H37Rv, or infected with Mtb H37Rv followed by Imatinib treatment (as explained in Figure 2B). Arrows indicate granuloma in gross lung morphology and regions of cellular aggregation in IHC sections. G, granuloma; FC, foam cells; N, focal center of degenerating neutrophils; AS, alveolar spaces. Low power (40×) and high power (400×) images of the same lung section are represented **(A)**. Granuloma scores of the lungs of PBS injected (*n* = 2), infected (*n* = 3), and inhibitor treated mice (*n* = 3) **(B)**. Morphology of spleens of infected and inhibitor treated mice **(C)**. Lung CFUs at Day 1 **(D)** and Day 28 **(E)** from Mtb H37Rv infected mice (*n* = 3, each group). Comparative analysis of lung **(F)** and splenic **(G)** weights, lung CFUs **(H)**, and splenic CFUs **(I)** (*n* = 5, each group) in infected and inhibitor treated mice. IHC, immunohistochemistry; ***P < 0.0001 (unpaired *t*-test).

The Mtb granuloma is a defining yet complex structure, and there remains much to be discovered regarding forces orchestrating this phenomenon (39). In an *in vivo* established TB model (Figure 2B), characteristic solid lesions were observed in greater numbers on the interstitium of infected mice lungs as compared to that in Mtb-infected mice treated with Imatinib (Figure 6A). Gross histopathology of the lungs revealed merely degenerating neutrophil containing foci in Imatinib treated mice (Figure 6A, lower panel), while untreated mice demonstrated active granulomatous structures with foam cells (Figure 6A, upper panel). Treatment with Imatinib resolved mycobacterial granuloma, as was evident from the granuloma scores (Figure 6B). Histopathology and scoring was also performed for PBS injected mice at the end of 56 days (Figures 6A,B). Mtb H37Rv infected mice displayed a discernable splenomegaly as compared to Imatinib treated mice (Figure 6C). Day 1 and Day 28 CFUs were assessed in the lungs of Mtb infected mice (Figures 6D,E). Lung weights (Figure 6F) and splenic weights (Figure 6G) of Imatinib treated mice were significantly lower than that of untreated mice, as were the reduced mycobacterial CFUs with Imatinib at Day 56 (Figures 6H,I). Our findings underscore an important role for c-Abl in the success of established mycobacterial infections.

### c-Abl Activated BMP Signaling Arbitrates TLR3 Driven Immune Signature

**Figure 7:**
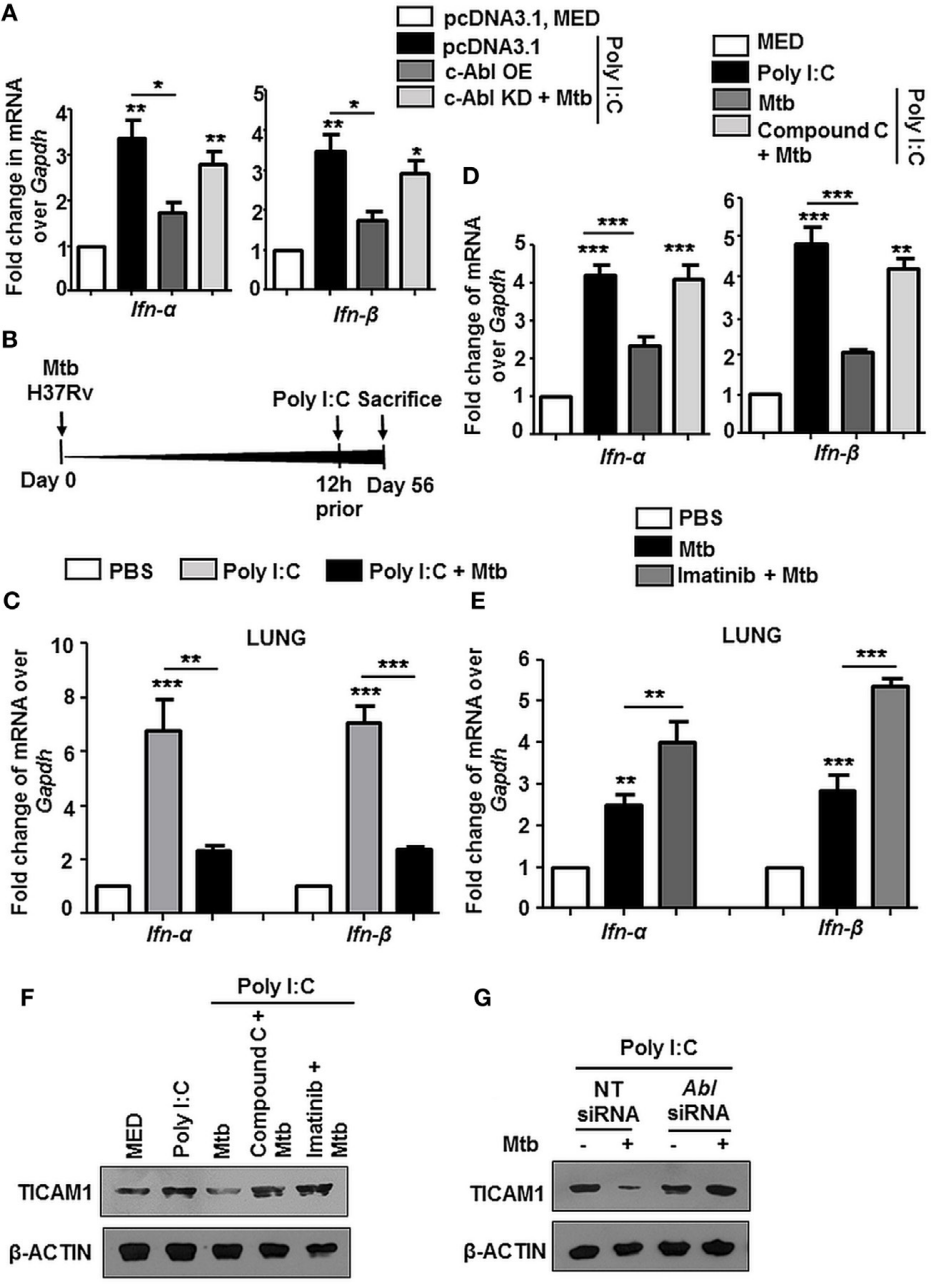
Mycobacteria coordinated c-Abl and bone morphogenesis protein (BMP) pathway dampen TLR3 responses. Transcript analysis of targets of activated TLR3 signaling *viz. Ifn-α* and *Ifn-β* was performed by quantitative real-time RT-PCR in the following instances **(A–E)**. RAW264.7 macrophages were transiently transfected with pcDNA3.1/c-Abl OE/c-Abl kinase dead (KD) constructs. These cells were pretreated with Poly I:C (1 h) followed by infection with Mtb. **(A)**. BALB/c mice (*n* = 6) were infected with 100 CFU of Mtb (aerosol) and injected with Poly I:C (10 mg/kg, intravenously) 12 h prior to sacrifice, i.e., Day 56 **(B)**. Lungs of these mice were assessed for *Ifn-α* and *Ifn-β* by quantitative real-time RT-PCR **(C)**. Mouse peritoneal macrophages were pretreated with Compound C (1 h) and Poly I:C (1 h), subsequently followed by Mtb infection as indicated **(D)** Analysis of *Ifn-α* and *Ifn-β* in lungs of mycobacteria-infected and mycobacteria-infected-Imatinib-treated mice (as explained in Figure 2B) **(E)**. Mouse peritoneal macrophages were pretreated with the indicated pharmacological inhibitors and Poly I:C (1 h) followed by Mtb infection where indicated. TICAM1 was assessed by immunoblotting **(F)**. RAW264.7 macrophages were transiently transfected with *Abl1* siRNA, pretreated with Poly I:C (1 h), infected with Mtb and assessed for TICAM1 by immunoblotting **(G)**. All *in vitro* Mtb infections were performed for 12 h, and data represent the mean ± SEM for five values from three independent experiments, *P < 0.05, **P < 0.005, ***P < 0.0001 (one-way ANOVA followed by Tukey’s multiple-comparisons test) and all blots are representative of three independent experiments.

Several instances report the cross-regulation among PRRs (40–42). We observed that Poly I:C triggered increase of Type I interferons (IFN-α and IFN-β) was inhibited substantially upon transient overexpression of c-Abl, and further, there was a rescue in their levels upon overexpression of c-Abl KD during Mtb infection (Figure 7A). Poly I:C mimics the effects of naturally occurring dsRNA and majority of its pleiotropic effects have been attributed to recognition by TLR3. In an *in vivo* model of TLR3-triggered events as depicted (Figure 7B), intravenous injection of Poly I:C resulted in a robust upregulation of IFN-α and IFN-β in lungs (Figure 7C) and spleens (Figure S3A in Supplementary Material) of these mice, while mice with established TB injected Poly I:C 12 h prior to sacrifice, manifested a fairly dampened IFN-α and IFN-β response. Further, we observed that this abrogation of TLR3 stimulated events was dependent on BMP signaling, as these events failed to unfold upon inhibition of BMP signaling using Compound C (Figure 7D). We found that type I interferon response was also elevated in mycobacteria-infected mice treated with Imatinib for 28 days, suggesting that treatment with Imatinib potentiated immune responses (Figure 7E; Figure S3B in Supplementary Material). In addition, Poly I:C induced TLR3 adaptor TICAM1 was downregulated during Mtb infection and this inhibition was lost if cells were pretreated with Compound C and Imatinib (Figure 7F) or siRNA directed against c-Abl (Figure 7G). Collectively, these results suggest that Mtb infection strongly inhibits Poly I:C induced inflammatory signature.

**Figure 8:**
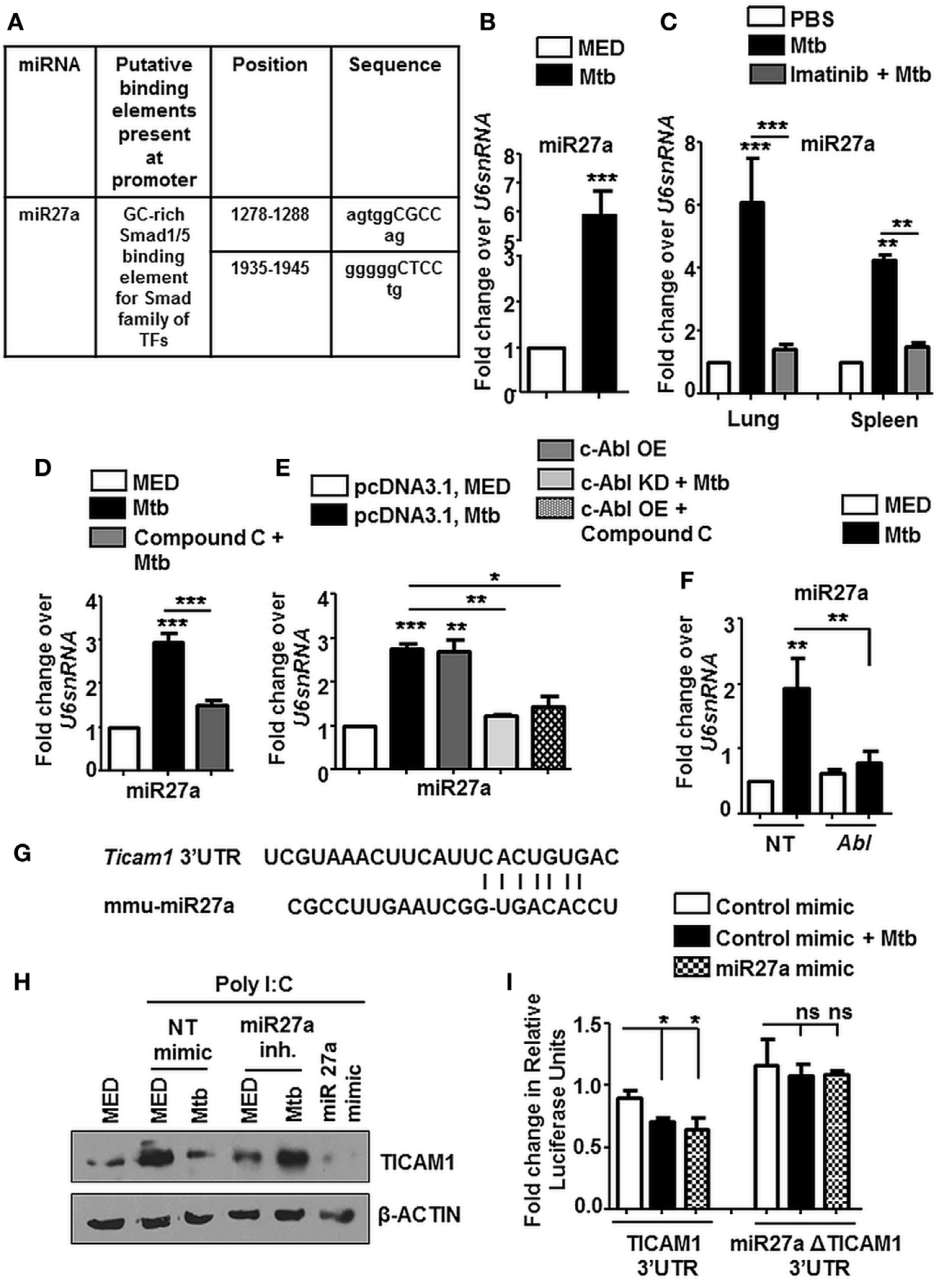
miR27a induced in response to mycobacterial infection, regulates TLR3 adaptor TICAM1. MatInspector assisted transcription factor binding site analysis of miR27a promoter **(A)**. Transcript levels of miR27a were assessed in each of the following instances by quantitative real-time RT-PCR **(B–F)**. Mouse peritoneal macrophages were infected with *Mycobacterium tuberculosis* (Mtb) H37Rv **(B)**. Total RNA was isolated from lungs and spleens **(C)** of Mtb infected mice followed by Imatinib treatment (as explained in Figure 2B). Mouse peritoneal macrophages were pretreated with Compound C followed by Mtb infection as indicated **(D)**. RAW264.7 macrophages were transiently transfected with pcDNA3.1/c-Abl OE/c-Abl kinase dead (KD) constructs, pretreated with Compound C (1 h) where indicated and infected with Mtb as shown **(E)**. RAW264.7 macrophages were transiently transfected with *Abl1* siRNA, subsequently followed by Mtb infection **(F)**. Putative miR27a binding sites in the 3′UTR of *Ticam1* **(G)**. RAW264.7 macrophages were transiently transfected with indicated miR mimic/inhibitor. These cells were pretreated with Poly I:C (1 h) and infected with Mtb where indicated. TICAM1 was assessed by immunoblotting **(H)**. RAW264.7 macrophages were transfected with wild-type or mutant (miR27aΔ) 3′UTR luciferase construct (TICAM1) along with miR27a mimic as indicated. 36 h postinfection, cells were infected with Mtb as indicated and luciferase assay was performed **(I)**. All *in vitro* Mtb infections were performed for 12 h, and data represent the mean ± SEM for five values from three independent experiments; ns, not significant; *P < 0.05, **P < 0.005, ***P < 0.0001 (one-way ANOVA followed by Tukey’s multiple-comparisons test) and all blots are representative of three independent experiments.

Tuberculosis infection is known to result in a deregulation of several host miRNA that are fundamental posttranscriptional regulators of gene expression. In this context, we observed Smad1/5 binding elements at the promoter of miR27a when assessed by MatInspector (Genomatix) (Figure 8A). These findings prompted us to assess the levels of miR27a during Mtb infection. miR27a was upregulated *in vitro* (Figure 8B) as well as *in vivo* in the lungs and spleen (Figure 8C) of Mtb infected mice. This upregulation was obstructed *in vivo* upon Imatinib treatment (Figure 8C) and *in vitro* upon pretreatment of macrophages with Compound C (Figure 8D). We validated our findings of induced miR27a through the overexpression of c-Abl WT and c-Abl KD during Mtb infection (Figure 8E). In addition, overexpression of c-Abl WT in the presence of Compound C failed to affect the expression of miR27a (Figure 8E), thus revealing a linear axis comprising c-Abl-BMP signaling which regulates miR27a. siRNA targeted against c-Abl prevented the Mtb induced upregulation of miR27a (Figure 8F), further underscoring the importance of the said sequence of events in directing the escalation of miR27a. Systematic bioinformatic analysis (Target Scan, miRanda, and miRWalk) identified target sites located at the residues spanning from 2,725–2,731 in the 3′UTR of TICAM1 as critical for its interaction with miR27a (Figure 8G). Enforced expression of miR27a inhibitor failed to bring about Mtb mediated negative regulation of TICAM1, while its mimic was sufficient to abrogate Poly I:C induced TICAM1 (Figure 8H). In addition, Mtb infection or transfection with miR27a mimic markedly reduced WT TICAM1 3′UTR luciferase activity. However, the reduction was not significant when mutant (for miR27a binding) TICAM1 3′UTR construct was utilized (Figure 8I). Altogether, Mtb responsive miR27a targeted Poly I:C induced signaling through the abrogation of TICAM1.

### DosR and Whib3 of Mycobacteria Are Important Regulators of c-Abl-BMP Signaling

**Figure 9:**
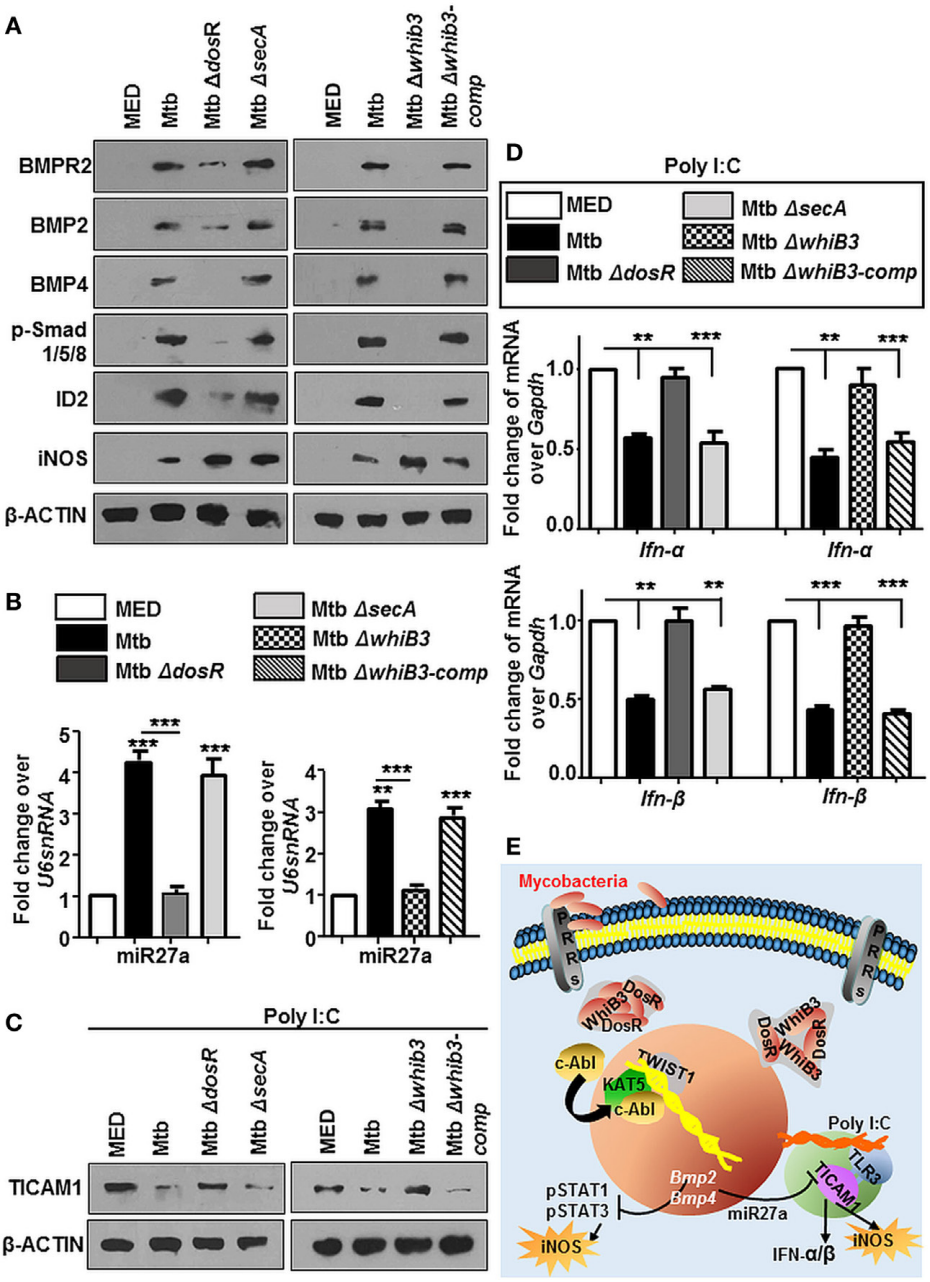
Mycobacterial WhiB3 and DosR depletion compromises bone morphogenesis protein (BMP) signaling and associated immune responses. Peritoneal macrophages from C57BL/6 mice were infected with mentioned strains of *Mycobacterium tuberculosis* (Mtb) [multiplicity of infection (MOI) 1:10] for 12 h in each of the following cases **(A–D)**. Hallmarks of activated BMP signaling and iNOS were analyzed by immunoblotting **(A)**. Transcript levels of miR27a were assessed by RT-PCR **(B)**. Mouse peritoneal macrophages were pretreated with Poly I:C (1 h) and infected with mentioned strains of Mtb as indicated. Total protein levels of TICAM1 were assessed by immunoblotting **(C)**. Transcript levels of *Ifn-α* and *Ifn-β* were assessed by quantitative real-time RT-PCR **(D)**. *Model*: Mtb result not only in the activation of cellular c-Abl but also in its nuclear translocation. In the nucleus, c-Abl associates with acetyltransferase KAT5 and recruits the transcription factor TWIST1 to BMP2/4 promoters resulting in its activation. Activated BMP signaling keeps Mtb stimulated host protective iNOS levels in check. It is also accompanied by a surge in the levels of miR27a that is a negative regulator of TICAM1, thus stifling TLR3-dependent innate responses **(E)**. All *in vitro* Mtb infections were performed for 12 h, and data represent the mean ± SEM for five values from three independent experiments, **P < 0.005, ***P < 0.0001 (one-way ANOVA followed by Tukey’s multiple-comparisons test) and all blots are representative of three independent experiments.

For many years, mycobacteria have exploited macrophages as their principle niche, which is also evident in this case of activated BMP pathway. To understand the mechanism of Mtb mediated modulation of BMP signaling, we employed Mtb mutants, namely, Mtb*ΔwhiB3, MtbΔdosR*, and Mtb*ΔsecA* that have been implicated in a global modulation of Mtb architecture. We observed that Mtb*ΔdosR* and Mtb*ΔwhiB3* failed to activate BMP signaling (Figure 9A) as well as miR27a (Figure 9B). SecA2 was not seen to play a role in these events. Yet, unsurprisingly, iNOS was elevated in all three Mtb mutants (Figure 9A). Further, we chose to focus on the role for DosR and WhiB3 in mycobacterial pathogenesis and found that macrophages infected with Mtb*ΔdosR* and Mtb*ΔwhiB3* also cross-regulated the TLR3 clade induced by Poly I:C (Figure 9C) including their effect on Type I interferons, *Ifn-α and Ifn-β* (Figure 9D). Altogether, our findings reveal novel roles for Mtb WhiB3 and DosR in regulating specific host signaling cascades (Figure 9E).

## Discussion

Several lines of investigation suggest the pathogenic modulation of c-Abl during infection. Emerging reports also suggest epigenetic changes associated with c-Abl (31, 43–45). Although it is clear that c-Abl regulates many phenomena in the host cells, information on signaling events and pathways associated with this key player remain scarce, particularly in context of Mtb infection. We found that c-Abl brings about an activation of BMP signaling during mycobacterial infection through its coordination with KAT5. Another factor often associated with BMP signaling is TWIST1. Interestingly, diacetylation of TWIST by KAT5 resulted in an active TWIST complex at the *Wnt5a* promoter in basal-like breast cancer (46). Thus, we propose a novel role of c-Abl mediated chromatin rewiring through KAT5 and TWIST1.

Reactive nitrogen intermediates comprising NO, NO_2_, and HNO_2_ have been demonstrated to play a vital role against mycobacteria in mice (47, 48). Our observation of c-Abl directed BMP signaling arbitrating iNOS suggested a mechanism for iNOS regulated control of Mtb infection under the aegis of Imatinib. While the levels of NOS in human alveolar macrophages or monocyte-derived macrophages remain debatable, it clearly does not imply a less vital role for NO in human immune defenses than in rodents (49).

This study is complementary to other screens corroborating the targeting of c-Abl during TB. While previous studies have laid stress on strains of mycobacteria such as *Mycobacterium marinum* and Mtb Erdman in mouse models, the role of Imatinib was majorly prophylactic. We chose to investigate the potential of Imatinib as a therapeutic agent in Mtb H37Rv induced TB at a dose of 66 mg/kg (7) injected immunoprecipitation every 2 days for 28 days. A considerable difference was observed in Imatinib treated mice, despite the low concentration, at the level of overall lung and spleen morphology, histopathology, and weight of lungs and spleen. Napier et al. observed an emergency myelopoiesis in *M. marinum* infected mice treated with Imatinib for 7 days (10). It was reflected in increased numbers of myeloid cell populations such as neutrophils, eosinophils, and monocytes in spleens of c-Abl inhibited mice. On the contrary, we observed reduced splenic and lung weights in mice with established TB, treated with Imatinib for 28 days, a likely indicator of abridged cellular infiltration. However, our study does not preclude the occurrence of such a myeloid expansion. In addition, a striking difference was observed in mycobacterial CFUs in lungs and more so in spleens of Imatinib treated mice at the end of 56 days. The diminished splenic CFU, in particular, is interesting and could represent either an enhanced mycobacterial control or a reduced pathogenic dissemination.

Incidences of lung cancer were approximately 11-fold higher among individuals suffering from TB as compared to non-TB subjects (50) and increasingly, it was observed that there exists a direct relationship between TB and cancer (51). However, very little is known about molecular mechanisms employed by mycobacteria to promote tumorigenesis. Previously, we have demonstrated a role for BCG in inhibiting TNF-α mediated clearance of tumors (52). The PRR, TLR3, was shown to participate in immune surveillance and result in tumor regression especially in lungs (53–55). Our study offers new insights into mycobacteria-activated c-Abl-BMP signaling abrogating TLR3 cascade, resulting in conditions that could plausibly fail to suppress tumor. Notwithstanding some reports suggest a role for TLR3 in tumor progression (56, 57). Classically, TLR3 is known to initiate a protective immune response against viral infections through the induction of Type I interferons (58). Thus, such a cross-regulation among PRRs (TLR2 and TLR3) causing subsequent downregulation of TLR3 and TICAM1 may result in weakened innate immunity against viral infections.

WhiB3 is a mycobacterial Fe-S cluster containing TF bearing the distinction of being the sole TF whose expression was found to be pH-responsive within macrophages (59). Further, a microarray of macrophages infected with Mtb*ΔwhiB3* revealed a number of upregulated innate immune cascades that were ordinarily suppressed by Mtb H37Rv (23). Mehta et al. also report Esx-1 as one of the important targets of WhiB3, thereby underscoring the loss of important TLR2-interacting components such as ESAT-6 in Mtb*ΔwhiB*. Recently, WhiB3 was demonstrated to facilitate the arrest of host cell-cycle and promote long-term persistence of mycobacteria (25). This study also reported the modulation of several innate immune genes, many of whose responses to mycobacteria remain to be explored. Further, the DosR regulon (co-regulating approximately 48 genes) is regarded as an important driver of mycobacterial dormancy (60). It has also been demonstrated that DosR mutants fail to persist or cause disease (24). Interestingly, Rv0079 and Rv3131encoded by DosR regulon were shown to interact with TLR2 (61, 62). Lastly, SecA2 of mycobacteria is an accessory secretion factor responsible for the release of detoxifying enzymes SodA and KatG (63). SecA2 is dispensable for growth, but plays a valuable role in Mtb virulence. Mice infected with Mtb*ΔsecA* revealed increased survival and a defective early growth phase possibly due to increased TNF-α, IL-6, RNI, and IFN-γ regulated MHC-II expression (64). Our findings of Mtb WhiB3 and DosR orchestrated manipulation of BMP signaling implicate a much larger role for global stresses such as NO, CO, and oxygen tension in dictating the outcome of mycobacterial infections.

In summary, our results associate an inflammatory host milieu in effecting the expression of mycobacterial factors such as DosR and WhiB3, which co-ordinate pathogenic evasion. We demonstrate here that treatment with Imatinib suppresses such pathogen directed deleterious responses. For over a decade now, FDA has approved Imatinib, which is now off the patent and therefore more affordable to the common man. Further, it is compatible with the first-line TB drugs apart from rifampicin, which can be overcome through its combination with rifabutin, making this host-targeted drug a potent adjunct. Collectively, our study adds new dimensions to the understanding of molecular cross-regulation in the host.

## Ethics Statement

All studies involving mice and virulent mycobacterial strains were carried out after the approval from the Institutional Ethics Committee for animal experimentation and from Institutional Biosafety Committee. The animal care and use protocol adhered to were approved by national guidelines of the Committee for the Purpose of Control and Supervision of Experiments on Animals (CPCSEA), Government of India.

## Author Contributions

Conceived and designed the study: KM and KNB Performed the experiments: KM, PP, and RR. Analyzed the data: KM, PP, and KNB. Contributed reagents: AS. Wrote the paper: KM and KNB.

## Conflict of Interest Statement

The authors declare that the research was conducted in the absence of any commercial or financial relationships that could be construed as a potential conflict of interest.

## References

[1] World Health Organization. Global tuberculosis report 2015. 20th ed (2015). 192 p.

[2] Mahon RN Hafner R. Immune cell regulatory pathways unexplored as host-directed therapeutic targets for *Mycobacterium tuberculosis*: an opportunity to apply precision medicine innovations to infectious diseases. Clin Infect Dis (2015) 61(Suppl 3):S200–16. 10.1093/cid/civ621 26409283 PMC4583576

[3] Wallis RS Hafner R. Advancing host-directed therapy for tuberculosis. Nat Rev Immunol (2015) 15:255–63. 10.1038/nri3813 25765201

[4] Zumla A Rao M Wallis RS Kaufmann SH Rustomjee R Mwaba P Host-directed therapies for infectious diseases: current status, recent progress, and future prospects. Lancet Infect Dis (2016) 16:e47–63. 10.1016/S1473-3099(16)00078-5 27036359 PMC7164794

[5] Hawn TR Matheson AI Maley SN Vandal O. Host-directed therapeutics for tuberculosis: can we harness the host? Microbiol Mol Biol Rev (2013) 77:608–27. 10.1128/MMBR.00032-13 24296574 PMC3973381

[6] Kundu M Pathak SK Kumawat K Basu S Chatterjee G Pathak S A TNF- and c-Cbl-dependent FLIP(S)-degradation pathway and its function in *Mycobacterium tuberculosis*-induced macrophage apoptosis. Nat Immunol (2009) 10:918–26. 10.1038/ni.1754 19597496

[7] Napier RJ Rafi W Cheruvu M Powell KR Zaunbrecher MA Bornmann W Imatinib-sensitive tyrosine kinases regulate mycobacterial pathogenesis and represent therapeutic targets against tuberculosis. Cell Host Microbe (2011) 10:475–85. 10.1016/j.chom.2011.09.010 22100163 PMC3222875

[8] Bruns H Stegelmann F Fabri M Dohner K Van Zandbergen G Wagner M Abelson tyrosine kinase controls phagosomal acidification required for killing of *Mycobacterium tuberculosis* in human macrophages. J Immunol (2012) 189:4069–78. 10.4049/jimmunol.1201538 22988030 PMC3684563

[9] Stanley SA Barczak AK Silvis MR Luo SS Sogi K Vokes M Identification of host-targeted small molecules that restrict intracellular *Mycobacterium tuberculosis* growth. PLoS Pathog (2014) 10:e1003946. 10.1371/journal.ppat.1003946 24586159 PMC3930586

[10] Napier RJ Norris BA Swimm A Giver CR Harris WA Laval J Low doses of imatinib induce myelopoiesis and enhance host anti-microbial immunity. PLoS Pathog (2015) 11:e1004770. 10.1371/journal.ppat.1004770 25822986 PMC4379053

[11] Woodring PJ Litwack ED O’Leary DD Lucero GR Wang JY Hunter T. Modulation of the F-actin cytoskeleton by c-Abl tyrosine kinase in cell spreading and neurite extension. J Cell Biol (2002) 156:879–92. 10.1083/jcb.200110014 11864995 PMC2173320

[12] Meltser V Ben-Yehoyada M Shaul Y c-Abl tyrosine kinase in the DNA damage response: cell death and more. Cell Death Differ (2011) 18:2–4. 10.1038/cdd.2010.132 21151157 PMC3131870

[13] Yogalingam G Pendergast AM. Abl kinases regulate autophagy by promoting the trafficking and function of lysosomal components. J Biol Chem (2008) 283:35941–53. 10.1074/jbc.M804543200 18945674 PMC2602914

[14] Kua HY Liu H Leong WF Li L Jia D Ma G c-Abl promotes osteoblast expansion by differentially regulating canonical and non-canonical BMP pathways and p16INK4a expression. Nat Cell Biol (2012) 14:727–37. 10.1038/ncb2528 22729085 PMC8265173

[15] Ghosh-Choudhury N Mandal CC Das F Ganapathy S Ahuja S Ghosh Choudhury G. c-Abl-dependent molecular circuitry involving Smad5 and phosphatidylinositol 3-kinase regulates bone morphogenetic protein-2-induced osteogenesis. J Biol Chem (2013) 288:24503–17. 10.1074/jbc.M113.455733 23821550 PMC3750149

[16] Andreu N Phelan J De Sessions PF Cliff JM Clark TG Hibberd ML. Primary macrophages and J774 cells respond differently to infection with *Mycobacterium tuberculosis*. Sci Rep (2017) 7:42225. 10.1038/srep42225 28176867 PMC5296737

[17] Das B Kashino SS Pulu I Kalita D Swami V Yeger H CD271(+) bone marrow mesenchymal stem cells may provide a niche for dormant *Mycobacterium tuberculosis*. Sci Transl Med (2013) 5:170ra113. 10.1126/scitranslmed.3004912 23363977 PMC3616630

[18] Garg RK Somvanshi DS. Spinal tuberculosis: a review. J Spinal Cord Med (2011) 34:440–54. 10.1179/2045772311Y.0000000023 22118251 PMC3184481

[19] Pigrau-Serrallach C Rodriguez-Pardo D. Bone and joint tuberculosis. Eur Spine J (2013) 22(Suppl 4):556–66. 10.1007/s00586-012-2331-y 22711012 PMC3691411

[20] Bleuming SA Kodach LL Garcia Leon MJ Richel DJ Peppelenbosch MP Reitsma PH Altered bone morphogenetic protein signalling in the *Helicobacter pylori*-infected stomach. J Pathol (2006) 209:190–7. 10.1002/path.1976 16550632

[21] Beckham JD Tuttle K Tyler KL. Reovirus activates transforming growth factor beta and bone morphogenetic protein signaling pathways in the central nervous system that contribute to neuronal survival following infection. J Virol (2009) 83:5035–45. 10.1128/JVI.02433-08 19279118 PMC2682065

[22] Miyazono K Kamiya Y Morikawa M. Bone morphogenetic protein receptors and signal transduction. J Biochem (2010) 147:35–51. 10.1093/jb/mvp148 19762341

[23] Mehta M Rajmani RS Singh A. *Mycobacterium tuberculosis* WhiB3 responds to vacuolar pH-induced changes in mycothiol redox potential to modulate phagosomal maturation and virulence. J Biol Chem (2016) 291:2888–903. 10.1074/jbc.M115.684597 26637353 PMC4742752

[24] Mehra S Foreman TW Didier PJ Ahsan MH Hudock TA Kissee R The DosR regulon modulates adaptive immunity and is essential for *Mycobacterium tuberculosis* persistence. Am J Respir Crit Care Med (2015) 191:1185–96. 10.1164/rccm.201408-1502OC 25730547 PMC4451619

[25] Cumming BM Rahman MA Lamprecht DA Rohde KH Saini V Adamson JH *Mycobacterium tuberculosis* arrests host cycle at the G1/S transition to establish long term infection. PLoS Pathog (2017) 13:e1006389. 10.1371/journal.ppat.1006389 28542477 PMC5456404

[26] Gopalakrishnan A Salgame P. Toll-like receptor 2 in host defense against *Mycobacterium tuberculosis*: to be or not to be-that is the question. Curr Opin Immunol (2016) 42:76–82. 10.1016/j.coi.2016.06.003 27326654 PMC5086274

[27] Buchdunger E Cioffi CL Law N Stover D Ohno-Jones S Druker BJ Abl protein-tyrosine kinase inhibitor STI571 inhibits in vitro signal transduction mediated by c-kit and platelet-derived growth factor receptors. J Pharmacol Exp Ther (2000) 295:139–45. 10991971

[28] Ando M Murakami Y Kojima F Endo H Kitasato H Hashimoto A Retrovirally introduced prostaglandin D2 synthase suppresses lung injury induced by bleomycin. Am J Respir Cell Mol Biol (2003) 28:582–91. 10.1165/rcmb.2002-0162OC 12707014

[29] Deininger M Buchdunger E Druker BJ. The development of imatinib as a therapeutic agent for chronic myeloid leukemia. Blood (2005) 105:2640–53. 10.1182/blood-2004-08-3097 15618470

[30] Jing Y Wang M Tang W Qi T Gu C Hao S c-Abl tyrosine kinase activates p21 transcription via interaction with p53. J Biochem (2007) 141:621–6. 10.1093/jb/mvm068 17339230

[31] Kaidi A Jackson SP. KAT5 tyrosine phosphorylation couples chromatin sensing to ATM signalling. Nature (2013) 498:70–4. 10.1038/nature12201 23708966 PMC3859897

[32] Reinhold MI Kapadia RM Liao Z Naski MC. The Wnt-inducible transcription factor Twist1 inhibits chondrogenesis. J Biol Chem (2006) 281:1381–8. 10.1074/jbc.M504875200 16293629

[33] Hayashi M Nimura K Kashiwagi K Harada T Takaoka K Kato H Comparative roles of Twist-1 and Id1 in transcriptional regulation by BMP signaling. J Cell Sci (2007) 120:1350–7. 10.1242/jcs.000067 17374642

[34] Quarto N Senarath-Yapa K Renda A Longaker MT. TWIST1 silencing enhances in vitro and in vivo osteogenic differentiation of human adipose-derived stem cells by triggering activation of BMP-ERK/FGF signaling and TAZ upregulation. Stem Cells (2015) 33:833–47. 10.1002/stem.1907 25446627 PMC5720150

[35] MacMicking JD North RJ Lacourse R Mudgett JS Shah SK Nathan CF Identification of nitric oxide synthase as a protective locus against tuberculosis. Proc Natl Acad Sci U S A (1997) 94:5243–8. 10.1073/pnas.94.10.5243 9144222 PMC24663

[36] Yang CS Shin DM Kim KH Lee ZW Lee CH Park SG NADPH oxidase 2 interaction with TLR2 is required for efficient innate immune responses to mycobacteria via cathelicidin expression. J Immunol (2009) 182:3696–705. 10.4049/jimmunol.0802217 19265148

[37] Yu PB Hong CC Sachidanandan C Babitt JL Deng DY Hoyng SA Dorsomorphin inhibits BMP signals required for embryogenesis and iron metabolism. Nat Chem Biol (2008) 4:33–41. 10.1038/nchembio.2007.54 18026094 PMC2727650

[38] Narayana Y Balaji KN. NOTCH1 up-regulation and signaling involved in *Mycobacterium bovis* BCG-induced SOCS3 expression in macrophages. J Biol Chem (2008) 283:12501–11. 10.1074/jbc.M709960200 18332140

[39] Martin CJ Carey AF Fortune SM. A bug’s life in the granuloma. Semin Immunopathol (2016) 38:213–20. 10.1007/s00281-015-0533-1 26577238 PMC4834868

[40] Jiang W Sun R Wei H Tian Z. Toll-like receptor 3 ligand attenuates LPS-induced liver injury by down-regulation of toll-like receptor 4 expression on macrophages. Proc Natl Acad Sci U S A (2005) 102:17077–82. 10.1073/pnas.0504570102 16287979 PMC1287976

[41] Kenny EF Talbot S Gong M Golenbock DT Bryant CE O’Neill LA. MyD88 adaptor-like is not essential for TLR2 signaling and inhibits signaling by TLR3. J Immunol (2009) 183:3642–51. 10.4049/jimmunol.0901140 19717524

[42] Trinath J Holla S Mahadik K Prakhar P Singh V Balaji KN. The WNT signaling pathway contributes to dectin-1-dependent inhibition of toll-like receptor-induced inflammatory signature. Mol Cell Biol (2014) 34:4301–14. 10.1128/MCB.00641-14 25246634 PMC4248746

[43] Aoyama K Fukumoto Y Ishibashi K Kubota S Morinaga T Horiike Y Nuclear c-Abl-mediated tyrosine phosphorylation induces chromatin structural changes through histone modifications that include H4K16 hypoacetylation. Exp Cell Res (2011) 317:2874–903. 10.1016/j.yexcr.2011.09.013 22001646

[44] Gonzalez-Zuniga M Contreras PS Estrada LD Chamorro D Villagra A Zanlungo S c-Abl stabilizes HDAC2 levels by tyrosine phosphorylation repressing neuronal gene expression in Alzheimer’s disease. Mol Cell (2014) 56:163–73. 10.1016/j.molcel.2014.08.013 25219501

[45] Aoyama K Yamaguchi N Yuki R Morii M Kubota S Hirata K c-Abl induces stabilization of histone deacetylase 1 (HDAC1) in a kinase activity-dependent manner. Cell Biol Int (2015) 39:446–56. 10.1002/cbin.10413 25561363

[46] Shi J Wang Y Zeng L Wu Y Deng J Zhang Q Disrupting the interaction of BRD4 with diacetylated Twist suppresses tumorigenesis in basal-like breast cancer. Cancer Cell (2014) 25:210–25. 10.1016/j.ccr.2014.01.028 24525235 PMC4004960

[47] MacMicking J Xie QW Nathan C Nitric oxide and macrophage function. Annu Rev Immunol (1997) 15:323–50. 10.1146/annurev.immunol.15.1.323 9143691

[48] Mills CD Kincaid K Alt JM Heilman MJ Hill AM. M-1/M-2 macrophages and the Th1/Th2 paradigm. J Immunol (2000) 164:6166–73. 10.4049/jimmunol.164.12.6166 10843666

[49] Muijsers RB Ten Hacken NH Van Ark I Folkerts G Nijkamp FP Postma DS l-Arginine is not the limiting factor for nitric oxide synthesis by human alveolar macrophages in vitro. Eur Respir J (2001) 18:667–71. 10.1183/09031936.01.00101301 11716172

[50] Yu YH Liao CC Hsu WH Chen HJ Liao WC Muo CH Increased lung cancer risk among patients with pulmonary tuberculosis: a population cohort study. J Thorac Oncol (2011) 6:32–7. 10.1097/JTO.0b013e3181fb4fcc 21150470

[51] Wu CY Hu HY Pu CY Huang N Shen HC Li CP Pulmonary tuberculosis increases the risk of lung cancer: a population-based cohort study. Cancer (2011) 117:618–24. 10.1002/cncr.25616 20886634

[52] Holla S Ghorpade DS Singh V Bansal K Balaji KN *Mycobacterium bovis* BCG promotes tumor cell survival from tumor necrosis factor-alpha-induced apoptosis. Mol Cancer (2014) 13:210 10.1186/1476-4598-13-210 25208737 PMC4174669

[53] Chin AI Miyahira AK Covarrubias A Teague J Guo B Dempsey PW Toll-like receptor 3-mediated suppression of TRAMP prostate cancer shows the critical role of type I interferons in tumor immune surveillance. Cancer Res (2010) 70:2595–603. 10.1158/0008-5472.CAN-09-1162 20233880 PMC2995454

[54] Shime H Matsumoto M Oshiumi H Tanaka S Nakane A Iwakura Y Toll-like receptor 3 signaling converts tumor-supporting myeloid cells to tumoricidal effectors. Proc Natl Acad Sci U S A (2012) 109:2066–71. 10.1073/pnas.1113099109 22308357 PMC3277567

[55] Pradere JP Dapito DH Schwabe RF. The Yin and Yang of toll-like receptors in cancer. Oncogene (2014) 33:3485–95. 10.1038/onc.2013.302 23934186 PMC4059777

[56] Zhan Z Xie X Cao H Zhou X Zhang XD Fan H Autophagy facilitates TLR4- and TLR3-triggered migration and invasion of lung cancer cells through the promotion of TRAF6 ubiquitination. Autophagy (2014) 10:257–68. 10.4161/auto.27162 24321786 PMC5396095

[57] Liu Y Gu Y Han Y Zhang Q Jiang Z Zhang X Tumor exosomal RNAs promote lung pre-metastatic niche formation by activating alveolar epithelial TLR3 to recruit neutrophils. Cancer Cell (2016) 30:243–56. 10.1016/j.ccell.2016.06.021 27505671

[58] Noppert SJ Fitzgerald KA Hertzog PJ. The role of type I interferons in TLR responses. Immunol Cell Biol (2007) 85:446–57. 10.1038/sj.icb.7100099 17667935

[59] Banaiee N Jacobs WR Jr Ernst JD. Regulation of *Mycobacterium tuberculosis* whiB3 in the mouse lung and macrophages. Infect Immun (2006) 74:6449–57. 10.1128/IAI.00190-06 16923787 PMC1695489

[60] Chen T He L Deng W Xie J. The *Mycobacterium* DosR regulon structure and diversity revealed by comparative genomic analysis. J Cell Biochem (2013) 114:1–6. 10.1002/jcb.24302 22833514

[61] Kumar A Lewin A Rani PS Qureshi IA Devi S Majid M Dormancy associated translation inhibitor (DATIN/Rv0079) of *Mycobacterium tuberculosis* interacts with TLR2 and induces proinflammatory cytokine expression. Cytokine (2013) 64:258–64. 10.1016/j.cyto.2013.06.310 23819907

[62] Peddireddy V Doddam SN Qureshi IA Yerra P Ahmed N. A putative nitroreductase from the DosR regulon of *Mycobacterium tuberculosis* induces pro-inflammatory cytokine expression via TLR2 signaling pathway. Sci Rep (2016) 6:24535. 10.1038/srep24535 27094446 PMC4837367

[63] Braunstein M Espinosa BJ Chan J Belisle JT Jacobs WR Jr. SecA2 functions in the secretion of superoxide dismutase A and in the virulence of *Mycobacterium tuberculosis*. Mol Microbiol (2003) 48:453–64. 10.1046/j.1365-2958.2003.03438.x 12675804

[64] Kurtz S Mckinnon KP Runge MS Ting JP Braunstein M. The SecA2 secretion factor of *Mycobacterium tuberculosis* promotes growth in macrophages and inhibits the host immune response. Infect Immun (2006) 74:6855–64. 10.1128/IAI.01022-06 17030572 PMC1698048

