# Supplementary material for: c-Abl-TWIST1 Epigenetically Dysregulate Inflammatory Responses during Mycobacterial Infection by Co-Regulating Bone Morphogenesis Protein and miR27a
Source: Front Immunol. 2018 Feb 1;9:85. doi: 10.3389/fimmu.2018.00085 (PMC5799226; doi:10.3389/fimmu.2018.00085)
Supplement: Supplementary file 1 [file Presentation_1.PDF]

SUPPLEMENTARY MATERIAL

c-Abl-TWIST1 epigenetically dysregulate inflammatory responses during mycobacterial infection by co-regulating BMP and miR27a

Kasturi Mahadik[1], Praveen Prakhar[1], R. S. Rajmani[2], Amit Singh[2] and Kithiganahalli Narayanaswamy Balaji[1*]

*To whom correspondence should be addressed. E-mail address: balaji@iisc.ac.in

FIGURES AND LEGENDS

Figure S1

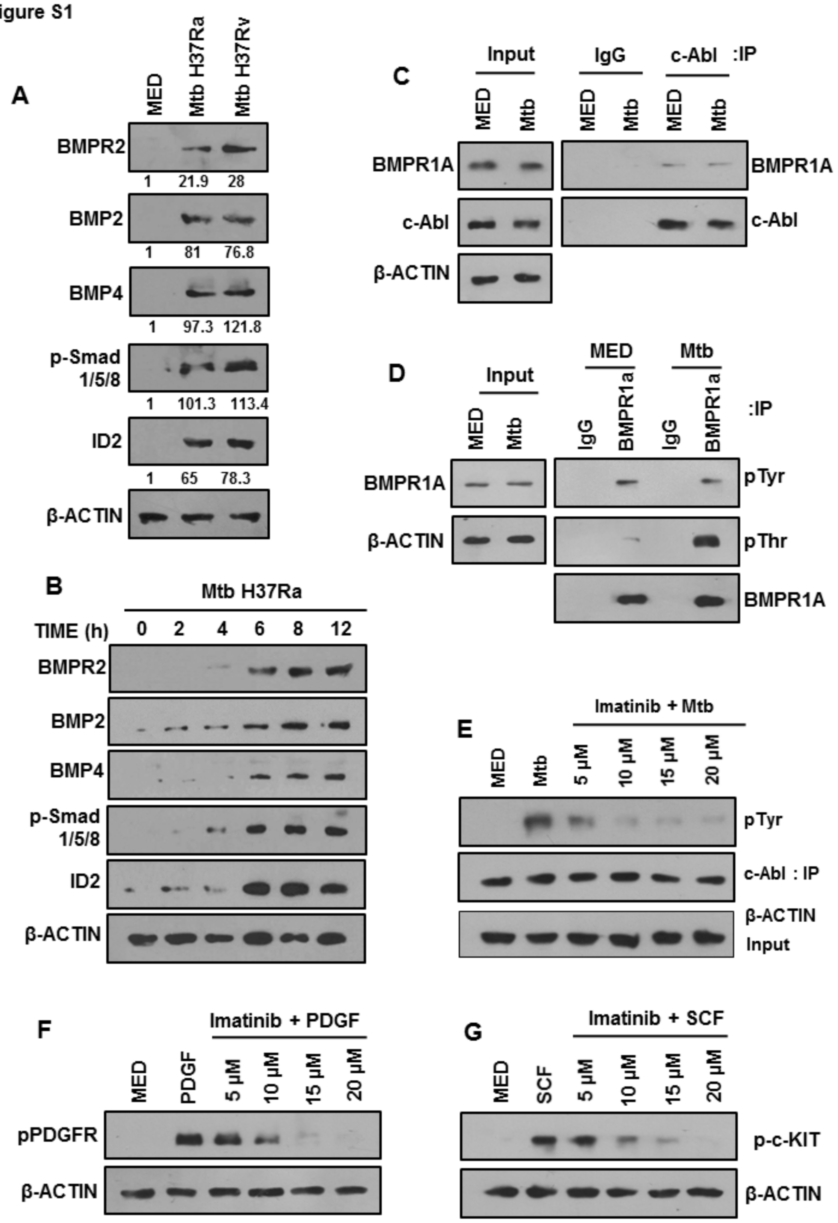

Figure S1. Mycobacteria activate BMP signaling and c-Abl. Densitometry analysis of immunoblot in Fig. 1B **(A).** Time kinetic analysis of hallmarks of BMP signaling activation upon infection with Mtb H37Ra was assessed by immunoblotting **(B).** c-Abl was immunoprecipitated in mouse peritoneal macrophages infected with Mtb (12h) and assessed for its interaction with BMPR1a by immunoblotting **(C).** BMPR1a was immunoprecipitated in mouse peritoneal macrophages infected with Mtb (12h) and assessed for its phosphorylated tyrosine and threonine status by immunoblotting **(D).** Mouse peritoneal macrophages were pre-treated with Imatinib (1h) at a range of concentrations as indicated, followed by Mtb infection (1h). c-Abl was immunoprecipitated and its phosphorylated tyrosine status was assessed by immunoblotting **(E).** NIH3T3 cells were pre-treated with Imatinib (1h) at a range of concentrations as indicated, followed by treatment with recombinant PDGF (10ng/ml) (1h) and phosphorylated tyrosine status of PDGFR was assessed by immunoblotting **(F).** K562 cells were pre-treated with Imatinib (1h) at a range of concentrations as indicated, followed by treatment with recombinant SCF (100ng/ml) (1h) and phosphorylated tyrosine status of c-KIT was assessed by immunoblotting **(G).** All blots are representative of 3 independent experiments.

Figure S2

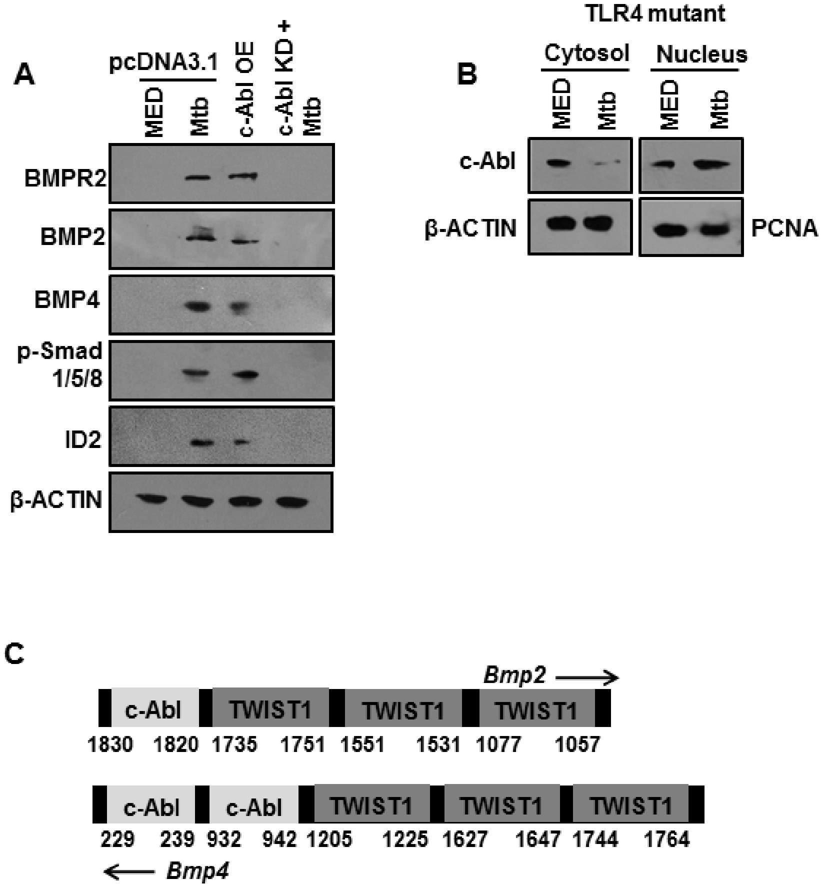

Figure S2. c-Abl orchestrates TLR4-independent activation of BMP signaling upon mycobacterial infection. RAW264.7 macrophages were transiently transfected with pcDNA3.1/c-Abl over-expression (OE) construct/c-Abl kinase dead (KD) construct followed by Mtb infection where indicated and assessed

for the activation of BMP signaling by immunoblotting **(A).** Nuclear-cytosolic fractionation of c-Abl in TLR4 mutant murine peritoneal macrophages was assessed by immunoblotting post Mtb infection **(B)**. MatInspector assisted TFBS analysis of BMP2 and BMP4 promoters **(C).** All *in vitro* Mtb infections were performed for 12h. All blots are representative of 3 independent experiments.

Figure S3

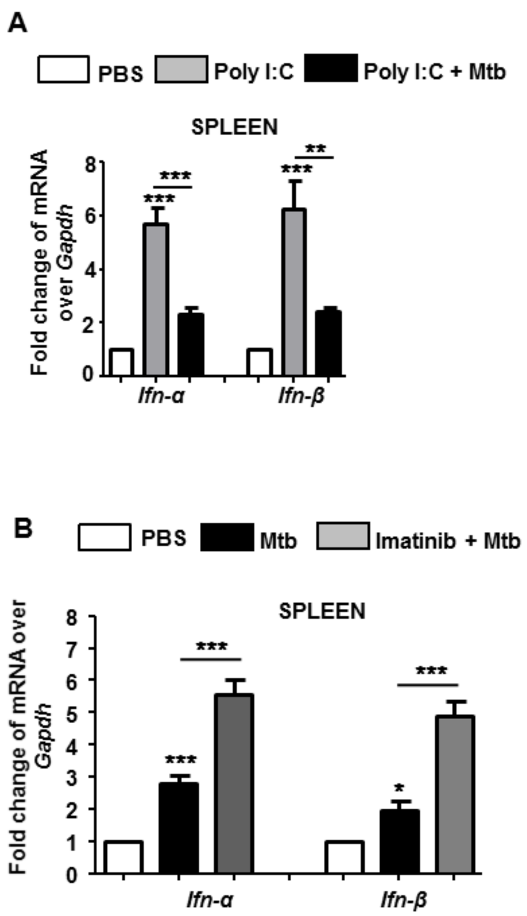

Figure S3. Imatinib regulates the levels of Type I interferons. Transcript analysis of *Ifn-α* and *Ifn-β* was performed by quantitative real time RT-PCR in spleens of Poly I:C injected mice with or without mycobacterial infection (as explained in Fig 7B) **(A)** and spleens of mycobacteria infected and mycobacteria-infected-Imatinib-treated mice (as explained in Fig. 2B) **(B).** *P<0.05, **P<0.005, ***P<0.0001 (One-way ANOVA followed by Tukey's multiple-comparisons test).
